# Improvement of Peak Integration in Capillary Electrophoresis: Reference Data Set No. 1

**DOI:** 10.1002/elps.70077

**Published:** 2026-03-11

**Authors:** Marlon Krompholz, Timothy Blanc, Huixin Lu, Patricia Christensen, Frédéric Ginot, Gábor Járvás, Trang D. Nguyen, Ashley Prout, Timothy Riehlman, Brian Wei, Andrei Hutanu, Steffen Kiessig, Knut Baumann, Cari E. Sänger‐van de Griend, Hermann Wätzig

**Affiliations:** ^1^ Institute of Medicinal and Pharmaceutical Chemistry Technische Universität Braunschweig Braunschweig Germany; ^2^ Biopharm Control Strategy, LLC. Branchburg New Jersey USA; ^3^ Centre for Oncology, Radiopharmaceuticals and Research Health Canada, Health Products and Food Branch, Biologic and Radiopharmaceutical Drugs Directorate Ottawa Ontario Canada; ^4^ Vaccines Analytical Research and Development Merck & Co., Inc. West Point Pennsylvania USA; ^5^ ADELIS SAS Labège France; ^6^ Research Institute of Biomolecular and Chemical Engineering University of Pannonia Veszprem Hungary; ^7^ Eli Lilly and Company Indianapolis Indiana USA; ^8^ Regeneron Pharmaceuticals Rensselaer New York USA; ^9^ BioProcess Analytics Sanofi Framingham Massachusetts USA; ^10^ ten23 health Basel Switzerland; ^11^ Kantisto BV Haaksbergen the Netherlands; ^12^ Department of Medicinal Chemistry Uppsala Universitet Uppsala Sweden

## Abstract

Capillary electrophoresis (CE) often provides superior separation of macromolecules such as monoclonal antibodies (mAbs), a major biopharmaceutical class, compared with liquid chromatography. However, electropherograms frequently exhibit complex baselines and peak shapes that are not reliably handled by integration algorithms designed for chromatographic data, and manual integration is often required. Many concepts have been proposed to improve peak integration, ranging from incremental algorithmic refinements and signal‐to‐noise (S/N)‐based approaches to artificial intelligence (AI)‐driven strategies, but objective performance comparisons are not possible without shared reference data sets and agreed peak limits. To address this gap, we initiated a multinational collaboration involving industrial and academic laboratories to create a comprehensive reference data set for CE peak integration. A total of 227 challenging and practically relevant electropherograms were collected from diverse applications, converted to a standardized format, and independently integrated by multiple experts. Using dedicated software tools and a structured consensus process, mutually accepted reference integration limits were established for each data set. These reference electropherograms, together with the underlying integration rules, are now made available to the scientific community. Analysis of the reference data set identified general principles for reliable peak integration, including the importance of standardized zoom levels and consistent handling of small peaks near the noise level. The data set provides a common foundation for benchmarking commercial chromatography data systems (CDS) and for developing and validating new algorithmic and AI‐based integration methods. We expect this work to speed up the development of practical, automated integration strategies for CE and that these core concepts can be applied to other separation techniques.

## 1 Introduction: Importance of Integration

Capillary electrophoresis (CE) frequently provides superior separation of macromolecules such as monoclonal antibodies (mAbs), a major class of biopharmaceuticals, compared with liquid chromatography (LC). This advantage arises because the lower diffusion coefficients of large molecules are beneficial for CE but detrimental for LC, thereby improving resolution and quantitation. However, electropherogram baselines and peak shapes pose challenges that are not adequately addressed by integration algorithms designed for chromatographic data, often necessitating complex, manual integration workflows [1]. In Good Manufacturing Practice (GMP) laboratories, robust and transparent peak integration is critical and has become a central focus of data integrity–oriented regulatory inspections. The combination of increasing use of CE, data systems optimized for chromatograms rather than electropherograms, and heightened regulatory scrutiny underscores the need for improved approaches to electropherogram integration. This need extends beyond GMP environments to R&D, clinical, and academic laboratories.

Over the past several decades, there has been little improvement in integration software, and references from the 20th century remain widely applicable [1]. Authoritative sources such as pharmacopeias, the World Health Organization (WHO), the Parenteral Drug Association (PDA), and the scientific literature provide guidance on chromatogram integration [1, 2, 3], but they do not address issues specific to electropherogram integration. These sources agree that companies should establish integration policies and Standard Operating Procedures (SOPs). As well, that all integration parameters must be recorded in detail, including slope sensitivity, smoothing factors, and time‐controlled events such as peak start and end times, as well as baseline correction settings. Additionally, it is highly recommended that analytical procedures (APs) should include illustrated instructions for each method [1]. These sources also emphasize that automatic integration should be used whenever possible, and that any manual integration must be documented and justified. Nevertheless, manual integration is still necessary in many cases because chromatography data systems (CDS) struggle to integrate electropherograms reliably.

Although automatic integration is preferred for its efficiency and objectivity, it is not always accurate. Therefore, manual integration should be allowed at the present time, with all settings and iterations documented, justified, and reviewed [1, 4].

The quality of the raw data plays a critical role in the success of peak integration [1]. For example, fluorescence detection often produces more stable baselines than ultraviolet (UV) detection because of lower background noise, which can substantially simplify integration. However, the higher sensitivity of fluorescence detection can also make very small peaks detectable, and in some cases, this increased sensitivity can complicate integration. In principle, CE would benefit from improved integration software regardless of the detector used. Because only a limited number of analytes are intrinsically fluorescent, relying solely on fluorescence detection is unlikely to be sufficient to improve peak integration, although it can contribute to better overall data quality when combined with other approaches.

Numerous strategies have been suggested to improve peak integration, ranging from incremental refinements of established algorithms to advanced approaches based on artificial intelligence (AI). These proposals may have found their way into commercially available software but have not been published in the scientific literature. To our knowledge, however, no approach has yet demonstrated objectively superior performance.

**FIGURE 1:**
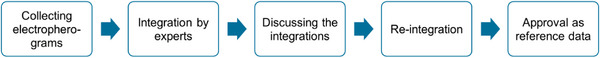
Workflow for determining reference data set no. 1 for CE peak integration.

Such a demonstration requires reference data sets that allow new algorithms to be compared directly with traditional methods. Reference data are essential for objectively assessing the performance of different integration software packages and for quantifying progress when existing tools are improved. Ideally, the reference integration limits would be proven correct by yielding 100% accurate peak areas. In practice, it is particularly difficult to demonstrate accuracy statistically, especially for complex electropherograms. We therefore adopted a pragmatic approach, assuming that integration limits that are broadly accepted by experts in the field can serve as the best available approximation to objectively correct integration at the present time. To this end, a multinational collaboration involving representatives from biopharmaceutical companies and universities was initiated (Table 1). Participants contributed electropherogram data sets, which were standardized in format and then integrated by expert teams. After reaching consensus on one or more integration strategies for each data set, the resulting consolidated reference data were compiled and are now being made available to the scientific community (Figure 1).

**TABLE 1:** List of all collaborators in alphabetical order.

Mostafa A. Atia, Timothy Blanc, **Tao Bo**, Patricia Christensen, **Miyuru de Silva**, Tara Enda, Gábor Járvás, Frédéric Ginot, Philip Hoang, Andrei Hutanu, Ryan Hylands, Steffen Kiessig, Marlon Krompholz, **Rick Linkous**, Huixin Lu, Jane Luo, **Will McElroy**, **Nathalie Montel**, Matthew Myers, Trang D Nguyen, Jeremy D. Osko, Ashley Prout, Timothy Riehlman, David Ripley, Cari Sänger‐van de Griend, Carlie M Schaeffer, Adam Sutton, Amanda Torelli, Ewoud van Tricht, **Carolina G. Vega**, **Yun Wang**, Hermann Wätzig, Brian Wei, Yuling Zhang

*Note*: The names of those who are not co‐authoring this article but provided this project with the necessary electropherograms or who participated in the integration or approval procedures as experts, thereby making a particularly valuable contribution, are printed in bold.

## 2 Experimental Procedures

To facilitate the discussion of integration limits among experts, three software packages were designed. These were the “Manual Integrator” (Supporting Information 6–8), the “Limit Comparator” (Supporting Information 9–11), and the “Limit Library” (Supporting Information 12–14).

The “Manual Integrator” was used by our experts to place markers for the start and end of each peak in an electropherogram. The resulting peak limits could be exported and sent to the organizers, enabling the collection of various integration approaches based on different types of expertise.

The “Limit Comparator” was used by the organizers to facilitate discussion about the collected integration approaches in terms of reasonability and reproducibility. The integration limits could be displayed simultaneously yet clearly distinguishable in the corresponding electropherograms, highlighting the differences in our experts’ approaches.

The “Limit Library” was used by our experts to review the proposed reference limits based on the outcome of the discussions and the integration rules derived from them. Experts could then either accept or reject the proposed limits, providing additional comments to explain their decision.

All three softwares can be downloaded and used in the form of .exe files, alongside the user manual, from Wiley's or TU Braunschweig's servers: https://cloud.tu‐braunschweig.de/s/4rNBxzdL7snS3Te.

### 2.1 Requirements for Reference Data Sets

**FIGURE 2:**
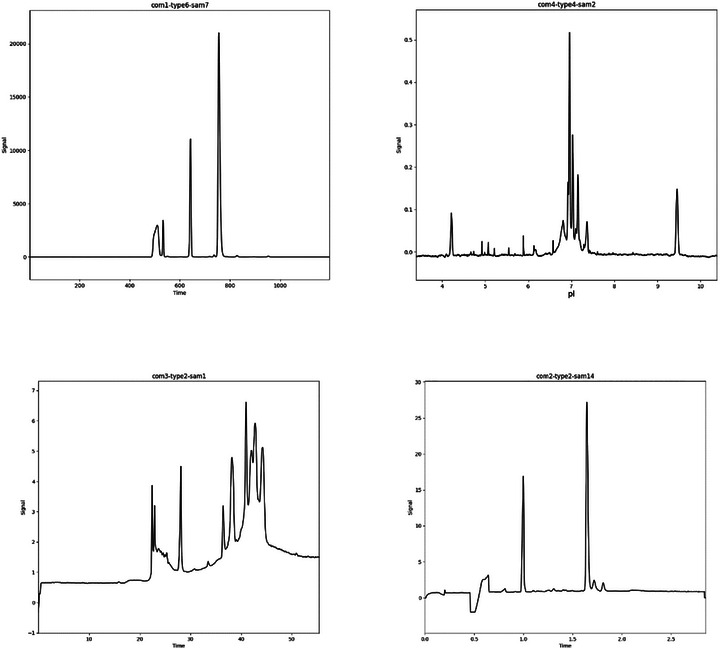
Selection of collected electropherograms that fulfill the desired requirements.

The reference data were selected to be reasonably complex, meaning that they were not trivial to integrate, and practically relevant, meaning that the complexity arose from genuine analytical challenges rather than from insufficient method optimization (Figure 2). Electropherograms from related samples were particularly encouraged in order to yield statistical statements by calculating standard deviations. Accordingly, the submitted data sets span a wide range of CE applications and collectively highlight multiple types of integration difficulty. Preferably, the data sets were originally integrated by the donors using their own CDS, which enables direct comparison with the consensus integrations generated in this study.

The .csv data format was chosen to facilitate data exchange and comparison. All participant data were submitted as .csv files or converted to this standard format. Each .csv file contained two columns with only the raw measurement data: time and response (e.g., absorbance). No additional information (metadata) was included in these files in order to ensure that the context of the data remains confidential and does not reveal any proprietary information. In addition to the CSV file submissions, there were additional files (e.g., .pdf or .pptx files) to provide contextual information. Alternatively, some contributors provided brief descriptions of the samples (e.g., monoclonal antibody) and the methods used (e.g., cIEF) separately by email, which enabled us to organize and categorize all submissions internally.

The collected data were intended to cover a broad spectrum of sample types and CE modalities. With respect to sample types, the data set includes RNA, reduced and non‐reduced proteins, various classes of mAbs, and multiple adeno‐associated virus serotypes. With respect to methods, the data set comprises CZE, cIEF, and CGE, each performed with either UV or fluorescence detection.

### 2.2 Data Collection Process

Previous efforts to improve peak integration have been hindered by limited opportunities to compare different approaches directly (see Section 1). Such comparisons require well‐defined reference data sets. At CE Pharm 2024 (CE in the Biotechnology & Pharmaceutical Industries: Symposium on the Practical Applications for the Analysis of Proteins, Nucleotides and Small Molecules), this need was examined in detail through presentations and subsequent discussions. As a result, a multinational collaboration involving industry, regulatory agencies, and universities was established (Table 1). In total, 227 data sets that met the agreed requirements (see Section 2.1) were collected and converted into a standardized format.

**FIGURE 3:**
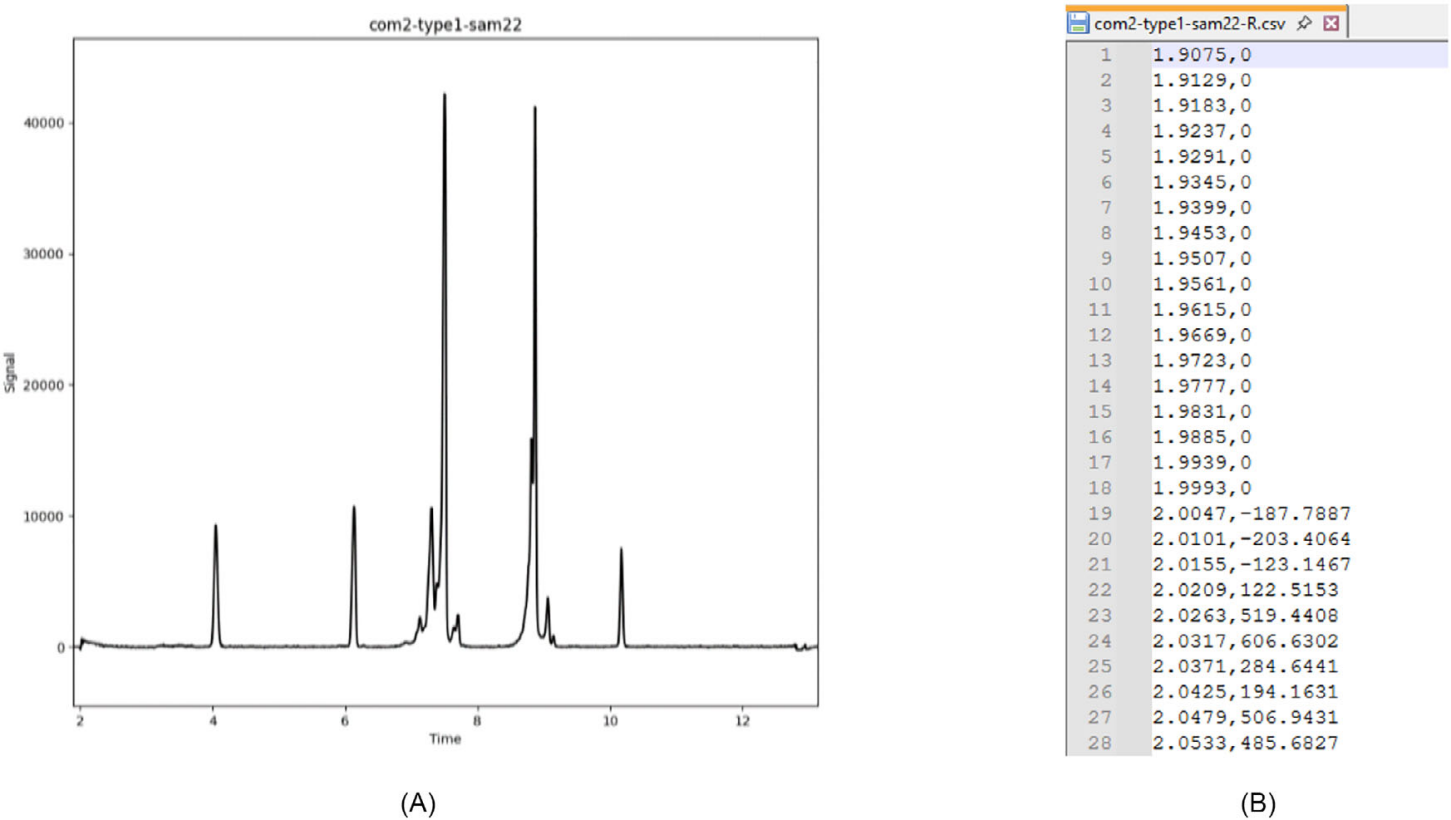
Representative electropherogram from reference data set no. 1 (A). It was named com2‐type1‐sam22‐R.csv because it was donated by company no. 2. It is the 22nd sample within the series of type1 from this company. R stands for “raw,” meaning not yet processed (no peak limits entered yet). The comma‐separated variable format .csv was chosen for its simplicity, as it can be read anywhere and therefore facilitates data exchange (B).

Each electropherogram was given a standardized yet unique file name according to the following scheme (Figure 3):

comX‐typeY‐samZ

- com: Company/Institution that generated/donated these data.
- type: Type of measurement (same analytical task, e.g., stability‐indicating assay or purity control of a certain analyte) that has been carried out.
- sam: Associated number of sample that has been measured.

This code structure keeps the data origin confidential while clearly showing which electropherograms are connected to each other, for example, within a series. Each electropherogram can be identified and referenced unambiguously by its associated file code.

*Examples regarding the code structure*:

*com1‐type1‐sam1 and com1‐type1‐sam2 are two samples from the same series measured by the same company. com1‐type1‐sam1 and com1‐type2‐sam1, on the other hand, are two different measurement types conducted by the same company. Samples with the same number but of different measurement types would not be related*.

New data, in the form of integration limits, were generated during the project, building on the collected electropherograms. To distinguish between the different types of data associated with the same electropherogram, the file code stem is complemented by either “‐R,” “‐P,” or “‐F,” indicating their respective data status.

- R: Raw data
  This ending belongs to the CSV file of the original electropherogram.
- P: Processed data
  This marks CSV files that contain peak boundaries determined by experts. The letter P will always be combined with the respective identifying number of the expert.
- F: Final reference data
  This indicates the final peak boundaries that will serve as reference data.

*Examples regarding the data status*:

*comX‐typeY‐samZ‐R: Electropherogram*.

*comX‐typeY‐samZ‐P1: Peak boundaries belonging to electropherogram comX‐typeY‐samZ‐R, proposed by expert with the identifying number 1*.

*comX‐typeY‐samZ‐P3: Peak boundaries belonging to electropherogram comX‐typeY‐samZ‐R, proposed by expert with the identifying number 3*.

*comX‐typeY‐samZ‐F: Final reference peak boundaries belonging to electropherogram comX‐typeY‐samZ‐R*.

All donated electropherograms are complex and difficult to integrate, but they originate from or are comparable to the practical work of a QC laboratory that uses CE. They often exhibit broad, asymmetric, and/or partly resolved peaks, fronting and tailing, and irregularities in the baseline. All 227 electropherograms from this set can be found under: https://cloud.tu‐braunschweig.de/s/4rNBxzdL7snS3Te.

### 2.3 Evaluation by Experts

To highlight the practical differences in how human experts interpret peak boundaries, all volunteer experts were asked to determine peak limits in a subset of the total reference electropherogram data set solely on the basis of their own professional judgment. To enable this, a custom software tool named “Manual Integrator” (see Section 2) was provided, which allowed experts to place markers for the start and end of each peak in an electropherogram. For those who were unable to use the custom software due to security restrictions, an alternative workflow was offered in which peak boundaries were marked in electropherograms using Excel.

The experts were divided into three groups. Each member of a given group received the same package, which contained a data set of 81–82 electropherograms in .csv format, the custom “Manual Integrator” software, and Excel files of the same electropherograms as an alternative way to mark peak boundaries. After the experts marked every start and end point for each peak they identified in a given electropherogram, the markers were either exported as a .csv file or saved within the corresponding Excel file and then returned to the authors. In total, 19 experts participated in this stage, resulting in at least 5–6 independent sets of integration limits generated by different experts for each electropherogram. Some electropherograms may provide more sets of integration limits as they have been given to all three groups or as duplicates of other electropherograms within one group (see Section 3.7).

In the next step, the exported peak boundary files were anonymized by renaming them with the corresponding expert identification number. This ensured that subsequent discussions were not influenced by the origin of the proposed peak boundaries, while still allowing integrations generated by the same individual to be identified and used for further statistical analysis of each expert's approach.

*Example*:

*comA‐typeB‐samC‐P1 and comX‐typeY‐samZ‐P1 are peak boundaries belonging to different electropherograms determined by the same person*.

The anonymized peak boundary proposals were then discussed in group meetings. Each one of the groups met separately to review and compare the different integration approaches used by its members.

To support moderation of the discussions, a second custom software tool named “Limit Comparator” (see Section 2) was provided. This tool allowed simultaneous display of all experts’ peak boundaries for a selected electropherogram, with each expert's boundaries shown in a different, randomly assigned color. Random assignment ensured that the discussion for each electropherogram focused on the boundaries themselves rather than sticking to previously favored approaches based on their color in the preceding electropherograms. To document consensus, the moderator could create a new set of peak boundaries within the loaded electropherogram and export the resulting limits.

The goal of the group meetings was to evaluate and compare different expert approaches to integration as well as to agree upon general integration rules that could lead to a reasonable integration approach.

### 2.4 Redefined Integration Limits

As a result of the group meetings, a guidance document containing several preliminary rules for manual integration was created (Supporting Information 5). These rules are based on the outcomes of the discussions.

For each electropherogram, all peaks were marked at their start and end again. This time, however, the determination of peak boundaries was carried out using the preliminary rules agreed by the groups, rather than relying on the expertise of the processing participant alone.

As a result, we obtained redefined integration limits for each electropherogram that are in line with the experts’ agreements from the group meetings.

### 2.5 Approval of the Experts

Once all the electropherograms had been re‐integrated, we asked all the experts who had contributed integration limits during the group stage to review the re‐integrated electropherograms and either approve or reject the proposed peak boundaries. A set of integration limits should be accepted if the expert considers it a reasonable approach to integration. Conversely, a set should be rejected if the expert considers any integration limit within it to be incorrectly placed.

To execute the review stage, each reviewer was provided with the aforementioned software named “Limit Library” (Section 2) that allowed them to review the assigned electropherograms and either accept or decline the proposed limits. Experts could also comment on individual markers and the entire marker data set for a given electropherogram, providing reasons for rejecting the boundaries and offering additional insights into the data. Each reviewer exported a report containing all approved and declined data sets, along with all comments, directly from the software. On the basis of the reviewers’ reports, all the mentioned peak boundaries were fine‐tuned to fulfill the requirements for final approval. In total, 8 experts participated in the review stage and were assigned 22–25 electropherograms each for approval.

**FIGURE 4:**
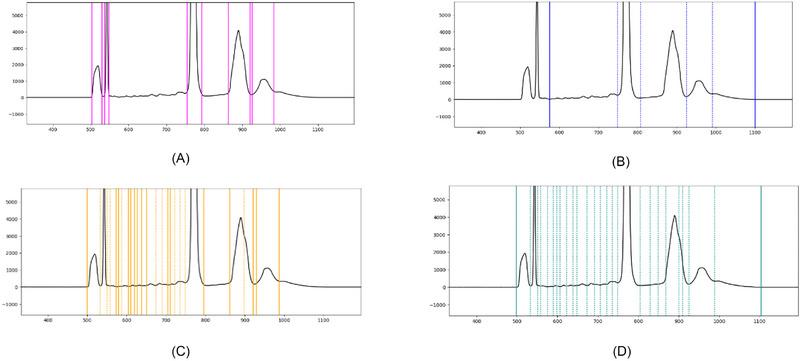
Examples of alternative integration approaches of the same data set conducted by different experts. Solid lines indicate the start or the end of a peak(‐group), whereas dotted lines represent integration limits shared by two peaks within a peak group. Integration limits from the same expert, and thus the same approach, are displayed in the same randomly assigned color, whereas integration limits from a different expert are displayed in a different color. Even though the approaches are clearly different from each other, none of them are necessarily wrong, as integration depends on the kind of information that should be gathered and therefore on the context of the analysis. (A) Rather minimalistic approach that focuses only on the main peaks of the electropherogram. (B) Approach that summarizes all the small peaks into one area, while ignoring peaks that do not seem to belong to the analyte of interest. (C) Approach that included those peaks that clearly differ from a potential baseline drift. (D) Rather elaborate approach that includes those peaks in the integration that clearly lie above the expected course of a non‐drifting baseline.

**FIGURE 5:**
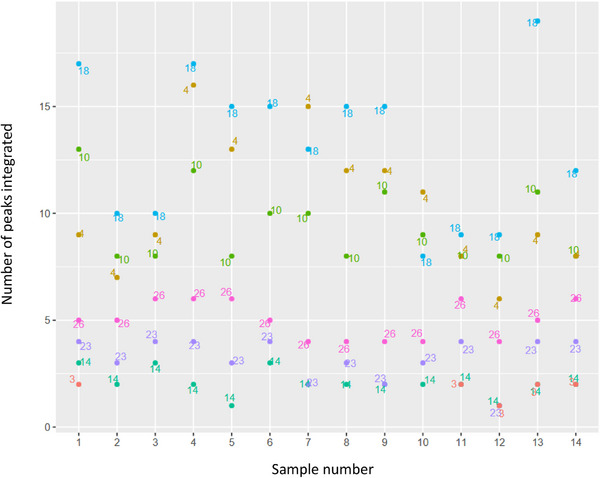
Number of peaks that each participant integrated into the com7‐type1 series (see https://cloud.tu‐braunschweig.de/s/4rNBxzdL7snS3Te). Each color and number next to the points represents a different participant, clearly demonstrating different approaches to integration. Some participants used a consistent integration method, with few peaks picked (3, 14, 23, 26), whereas others showed significantly more variation in the number of peaks picked across the series (4, 10, 18). This could indicate that participants 4, 10, and 18 integrated a greater number of controversial peaks and included them in some electropherograms while excluding them in others. Another possible explanation for this inconsistency is that they integrated small peaks that appeared only in some electropherograms, for example, those from samples with higher concentrations.

During this process, it became apparent that, even after extensive discussion, complete agreement among all participating experts on the integration limits could not be achieved. Expert judgments were clearly influenced by prior experience with specific applications, which is consistent with the common industry practice of training analysts and reviewers on particular APs. For example, in a CE‐SDS electropherogram, early migrating peaks are typically system peaks or peaks related to the sample matrix, and an analyst familiar with this application will generally not integrate these peaks, whereas someone unfamiliar with the method may be unable to make this distinction. Another example of alternative integration options that depend on the analytical context is shown in Figure 4, whereas Figure 5 illustrates the general variability of approaches taken by different experts.

Therefore, the aim of this study was not to determine the “true” peak limits, which is not realistically achievable, but rather to establish mutually agreed, practically appropriate limits. The resulting limits are intended to be reasonable in the sense that, when evaluated by an expert, none of the peak boundary decisions should be regarded as clearly incorrect.

Following the reviewers’ approval, we compared the integration limits for each electropherogram with those of other, similar electropherograms (e.g., from the same series) to ensure consistent determination of peak boundaries. To further confirm that the final limits were reasonable, we also compared them with the integration limits provided initially by the experts at the beginning of the project to avoid defining reference limits that no expert would realistically select.

Finally, we presented this work to all collaborators and at CE Pharm 2025 in Bethesda. In both settings, we obtained broad support from the audience, indicating that the proposed reference integration limits are generally acceptable to the participating scientific community. These integration limits, along with the underlying rules that define them, will be made available via Wiley's server and the TU Braunschweig website (https://cloud.tu‐braunschweig.de/s/4rNBxzdL7snS3Te). Both the final limits and the associated rules should be regarded as provisional and may be revised if further experience or analysis suggests more appropriate approaches.

**FIGURE 6:**
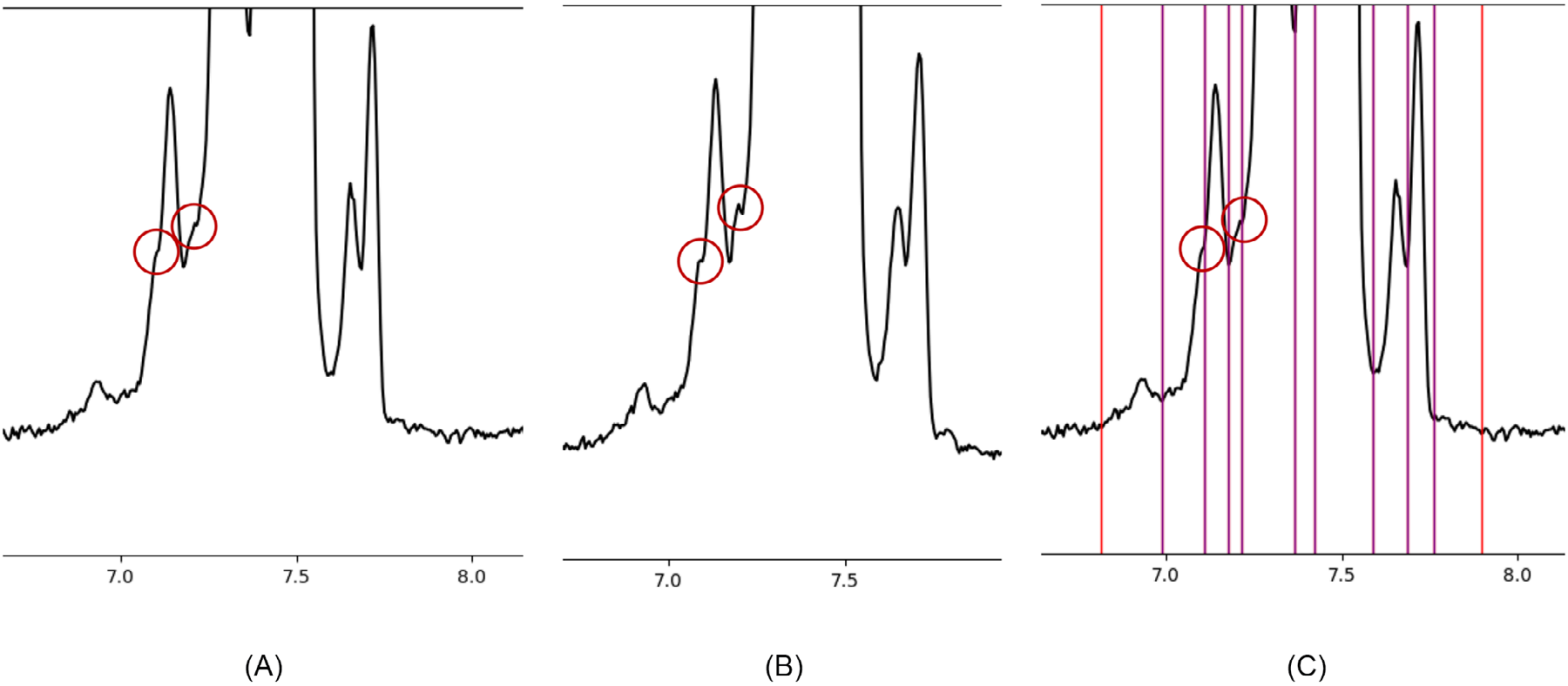
Borderline case as to whether a shoulder should be recognized or not. (A) Electropherogram with potential shoulders. (B) Another electropherogram from the same series with more distinct shoulders. (C) Final reference limits that have been chosen for the electropherogram from A. Red markers display the start or the end of a peak(‐group), whereas purple markers display shared integration limits within a peak group.

Even though a consensus was reached among the experts, borderline cases remain in which different interpretations are possible. One example is the assessment of shoulders (Figure 6). Some analysts maintain that a shoulder should be recognized only when a clear valley or plateau is present, whereas others consider a discernible deviation in the main peak's slope sufficient, particularly when a similar electropherogram shows a well‐defined shoulder at the same migration position.

It should be mentioned that by reaching a consensus, we do not claim to provide the most accurate integration limits. Further investigations are needed, and integration strategies should be optimized given the context of use.

## 3 Integration Guidelines

### 3.1 Zoom Level

**FIGURE 7:**
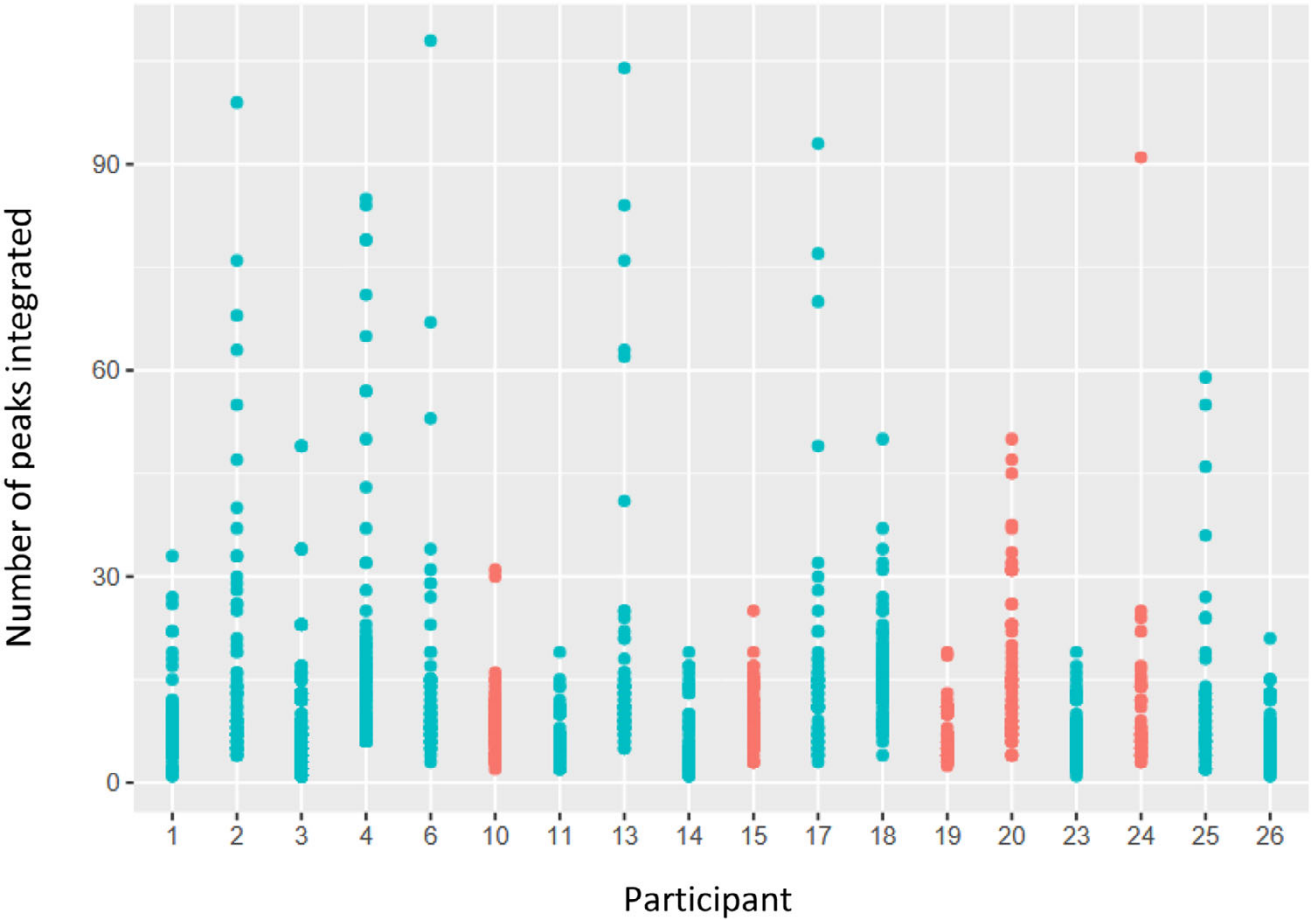
Number of peaks integrated by each participant, with each point representing a single electropherogram. Participants who used the “Manual Integrator” software are displayed in blue, whereas experts who used “Excel” for the determination of integration limits are displayed in red. Participants who used the “Manual Integrator” software tend to find more peaks possibly due to the higher zoom capabilities.

Discussions among the experts indicated that the zoom factor used during evaluation can substantially influence how peaks are integrated. This observation is supported by Figure 7, which shows that participants who used Excel to integrate the electropherograms tended to integrate fewer peaks than those who used the “Manual Integrator” software due to its enhanced “zoom” feature. It should be noted, however, that each participant's experience and the usability of the respective software are likely to have contributed to the inclusion or exclusion of small peaks. Nevertheless, these findings emphasize the need to document and, where possible, standardize the zoom level used whenever manual integration is performed or when manual or automatic integrations are reviewed.

It is advisable that integration software displays the zoom level numerically and allow numerical adjustment of the zoom factor. Whenever an integration limit is manually set or modified, the zoom level used should be recorded as metadata to ensure that the integration can be reproduced and verified during audits.

Furthermore, we recommend that integration software provides predefined zoom levels as practical viewing settings for individual electropherograms. These preset zoom levels can be used directly or serve as starting points for the analyst to adjust and identify the most appropriate zoom level for manual integration of a given electropherogram.

**FIGURE 8:**
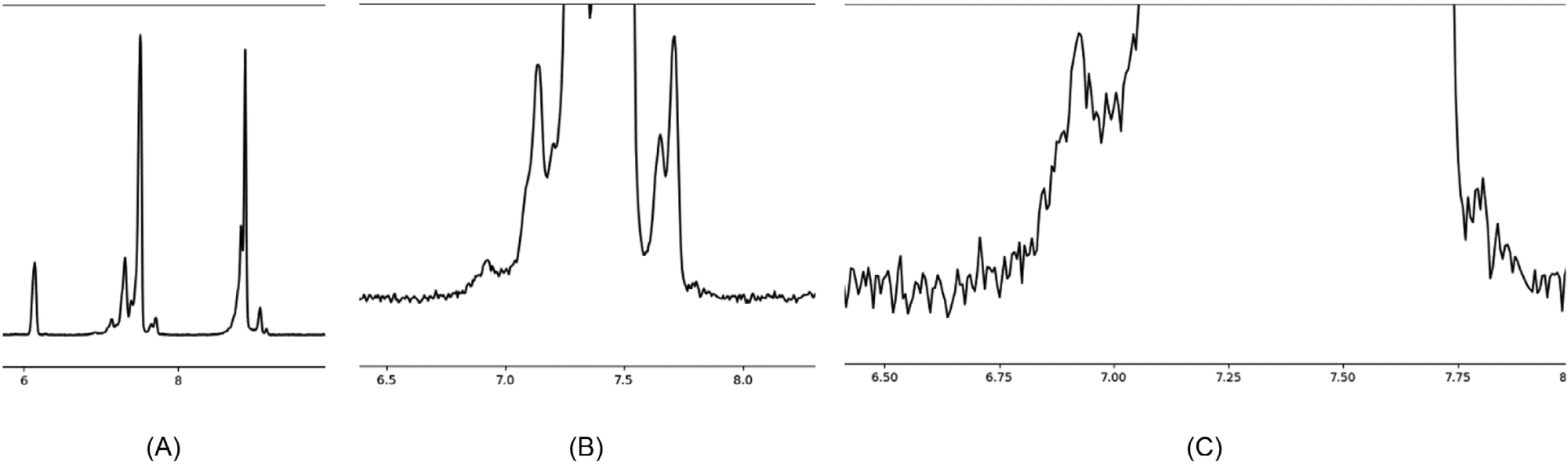
The final integration result depends heavily on the selected zoom level (see Section 3.1). For an (im)purity application, it is recommended to first view the complete electropherogram without zoom (A), then with preset 1 which includes a 15× magnification on the *y*‐axis (B), and finally with preset 2 which includes a 100× magnification on the *y*‐axis (C).

During our work, the following zoom factors have proven to be particularly suitable as presets (Table 2 and Figure 8).

**TABLE 2:** Reasonable zoom levels for appropriate manual integration.

	Zoom preset 1	Zoom preset 2	Calculation basis
Factor *X*‐axis	3	6	*x*_max_ − *x*_min_
Factor *Y*‐axis	15	100	*y*_max_ − *y*_min_

*Note*: Zoom preset 1 would show 1/3 of the original *x* span and 1/15 of the original y span, providing a moderate zoom. Preset zoom level 2 would show 1/6 of the *x* span and 1/100 of the *y* span, providing a highly detailed zoom. For specific applications, there might be better presets than the ones we suggest; however, our preset zoom levels are starting points to build upon. A quantitative assay would require a different zooming level than an impurity method.

Instead of basing the zoom level on the full axis span, it may be advantageous to use the signal‐to‐noise (S/N) ratio, as this is less affected by a single very intense peak in an electropherogram that also contains much smaller peaks. Prominent peaks can distort zoom levels calculated from the span, because the *y*‐maximum is always set by the highest intensity peak in the electropherogram. The potential and optimal implementation of an S/N ratio‐based zoom will be explored in future work.

### 3.2 Classification of Small Peaks

**FIGURE 9:**
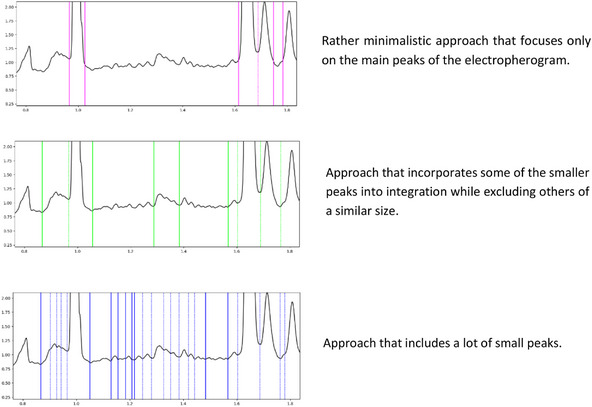
Examples of alternative integration approaches of the same data set conducted by different experts regarding a section of small peaks with slightly varying sizes.

Even when the zoom factor is set appropriately, accurately integrating small peaks remains challenging. This is illustrated in Figure 9. In this example, some small peaks are still sufficiently prominent to be recognized as peaks by all experts. However, there are also slightly smaller peaks of varying amplitudes, with only minor differences in height from one to the next, and finally, the smallest features appear indistinguishable from noise. In such cases, it is difficult to differentiate true peaks from background noise. This difficulty is one of the reasons why the experts were unable to agree on a single integration approach for these electropherograms (see Section 2.5).

In such situations, it is unrealistic to expect every decision to be correct. In some cases, noise will be misclassified as a peak, whereas in others, true peaks will be incorrectly treated as background noise. This problem is analogous to a statistical significance test. One must decide whether a signal can still be regarded as background noise, that is, not statistically different from zero (related to the probability of a type I, or *α*, error), or whether it is statistically significantly different from zero (related to the probability of a type II, or *β*, error). In both cases, there is a non‐zero probability of error. Our goal cannot be to eliminate these errors entirely but rather to reduce both types of error as much as possible.

As this decision problem is analogous to determining the limit of detection, we propose adopting an approach similar to that described in the European Pharmacopoeia [5], which explicitly considers both *α* and *β* errors. Specifically, a section of the baseline should be selected that lies as close as possible to the potential peaks of interest and has a length of approximately 20 times *h*_0.5_ of a main peak (where *h*_0.5_ denotes the peak width at half height). The peak‐to‐peak noise is then estimated from this baseline segment. Blank electropherograms are recommended to support the estimation of S/N ratios, if possible. All signals with an S/N ratio greater than 3 can be classified as peaks. At S/N = 3, the probability of misclassifying true peaks as noise or noise as peaks is approximately 7% [6], which we consider acceptable for this purpose.

### 3.3 Manual Integration Guidelines

The following guidelines were applied in this project to achieve mutually agreed integration limits. We consider them useful for developing SOPs for manual integration of purity methods. These guidelines can also be used to train analysts in manual integration, thereby reducing variability in integration practices. Training is likely to be more effective when supported by illustrative examples. An excerpt from our integration manual (given in the Supporting Information section) is provided in the following section, and the complete integration manual, including all example illustrations, is available in the Supporting Information section.

### 3.4 Integration Manual

Part of the scientific article “Improvement of peak integration in Capillary Electrophoresis: Reference Data Set No. 1” by

Marlon Krompholz^1^, Timothy Blanc^2^, Huixin Lu^3^, Patricia Christensen^6^, Frédéric Ginot^7^, Gábor Járvás^8^, Trang D. Nguyen^9^, Ashley Prout^6^, Timothy Riehlman^10^, Brian Wei^11^, Andrei Hutanu^12^, Steffen Kiessig^12^, Knut Baumann^1^, Cari E. Sänger‐van de Griend^1,4,5^, Hermann Wätzig^1^

Submitted to Electrophoresis in 2025.

#### 3.4.1 Appropriate Display in Three Steps

This manual describes the integration approach used in the Intercompany Collaboration (ref) and serves as a guide for future work with the reference data set. It also provides guidance for reviewers to assess whether integrations have been performed correctly.

The analyst determines the integration limits in electropherograms by successively applying different methods. Viewing electropherograms at various degrees of magnification (zoom levels) is a critical step in determining the appropriate integration limits (peak start and peak end) of electropherograms. When working at these various zoom levels, some basic rules (see below) should be followed.

Level 1: Full‐scale (no zoom)

View the electropherogram in full‐scale first. Set markers at the start and end of every clearly recognizable peak in the electropherogram.

Level 2: Moderate zoom (e.g., y‐scale x 15, x‐scale x 3)

Zoom in to obtain a more detailed view of the peak. Adjust the integration limits set at Level 1 as needed to improve their placement. If additional peaks become visible at this zoom level, set start and end markers for each new peak.

Level 3: Highly detailed zoom (e.g., y‐scale x 100, x‐scale 6)

Zoom‐in further to achieve a very detailed view of the peaks. Adjust integration limits according to the specific rules (see below).

See importance of the zoom

Moving progressively from low detail (Level 1) to very high detail (Level 3) helps the analyst maintain perspective and context when defining peak boundaries. At very high zoom, it becomes difficult to distinguish potential peaks from surrounding noise. Therefore, it is not recommended to start directly at Level 3; instead, the zoom level should be increased stepwise to preserve an overall view of the electropherogram.

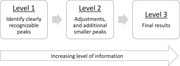

### 3.5 Rules for Peak and Peak Limit Determinations

#### 3.5.1 Basic Rules

- When peaks are obvious and definitively emerge from the underlying baseline, they should be marked at the beginning and end of this emergence. Whenever there is any uncertainty as to whether an area represents a peak or part of the baseline, the area in question will be considered as a peak if it meets either of the following criteria:
  - It exceeds an S/N ratio of 3 relative to the surrounding noise (see Example 2.1) or
  - the peak is present at higher levels in related samples (e.g., a later stability timepoint) from the same series or not present in blank injections associated with the sample analysis (see Example 2.2).
- When two peaks merge because of an insufficient baseline separation, they always share a marker. The shared marker represents both the end of the first peak and the beginning of the following peak (see Example 2.3).
- It is essential to always consider the baseline noise. If two peaks are unresolved, but the section between them shows a noise level consistent with the rest of the electropherogram, the two peaks will not be considered a peak group and therefore will not share a marker (see Example 2.4).
- If a section of the electropherogram shows an obvious noise pattern but clearly lays above the expected baseline, this section will be considered as a peak (see Example 2.5).
- A shoulder is an unresolved peak on the leading or trailing edge of a larger peak. To be treated as a shoulder, the signal must show either
  - a local minimum relative to the main peak, or
  - a distinct inflection region on the main peak where the local slope is approximately zero.
  - If no such local minimum or zero‐slope inflection is present, or if this feature could reasonably be interpreted as baseline noise, the region is not considered a shoulder unless comparison with other injections in the same series clearly confirms a reproducible feature at the same migration position (see Example 2.6).
- Shoulders do not need to meet the requirement of exceeding a signal‐to‐noise ratio of 3 to be considered.
- Negative peaks are taken into account and will therefore be marked in compliance with the other basic rules (see Example 2.7).
- Peaks that can be clearly identified as system peaks and are therefore not related to the sample will not be included in the integration. The presence of system peaks can be confirmed by blank injections without analyte (see Example 2.8).

#### 3.5.2 Specific Rules on Where to Place the Integration Limits

General

**Table:** 

1.	Does the peak emerge from a completely flat region of the baseline (no slope)?
	Yes → Beginning/end of slope.
	See Example 3.1
	No → Apply any of the following rules.
2.	Is there a peak immediately before the considered peak (unresolved peaks)?
	Yes → Valley (local minimum) between the peaks.
	See Example 3.2
	If the valley is overlaid by noise, apply Rule 3.9 or Rule 3.10.
	No → Is there a local minimum immediately before the main increase?
	Yes → Local minimum.
	See Example 3.3
	No → Is there an inflection point immediately before the main increase?
	Yes → Inflection point.
	See Example 3.4
	No → Point where the increase significantly changes its rate.
	See Example 3.5
3.	Is there a peak immediately after the considered peak (unresolved peaks)?
	Yes → Valley (local minimum) between the peaks.
	See Example 3.2
	If the valley is overlaid by noise, apply Rule 3.9 or Rule 3.10.
	No → Is there local minimum immediately after the main decrease?
	Yes → Local minimum.
	See Example 3.6
	No → Is there an inflection point immediately after the main decrease?
	Yes → Inflection point.
	See Example 3.7
	No → Point where the decrease significantly changes its rate.
	See Example 3.8
(…)	

### 3.6 First Results From the Data Set

#### 3.6.1 Setting Peak Limits and Baselines: The Importance of Context

Peak integration often relates to the context. Does an area belong to the matrix or to the analyte? Knowledge of the analyte's origin, composition, and matrix is essential in practical cases. Blank electropherograms are indispensable if significant matrix effects are expected [1].

**FIGURE 10:**
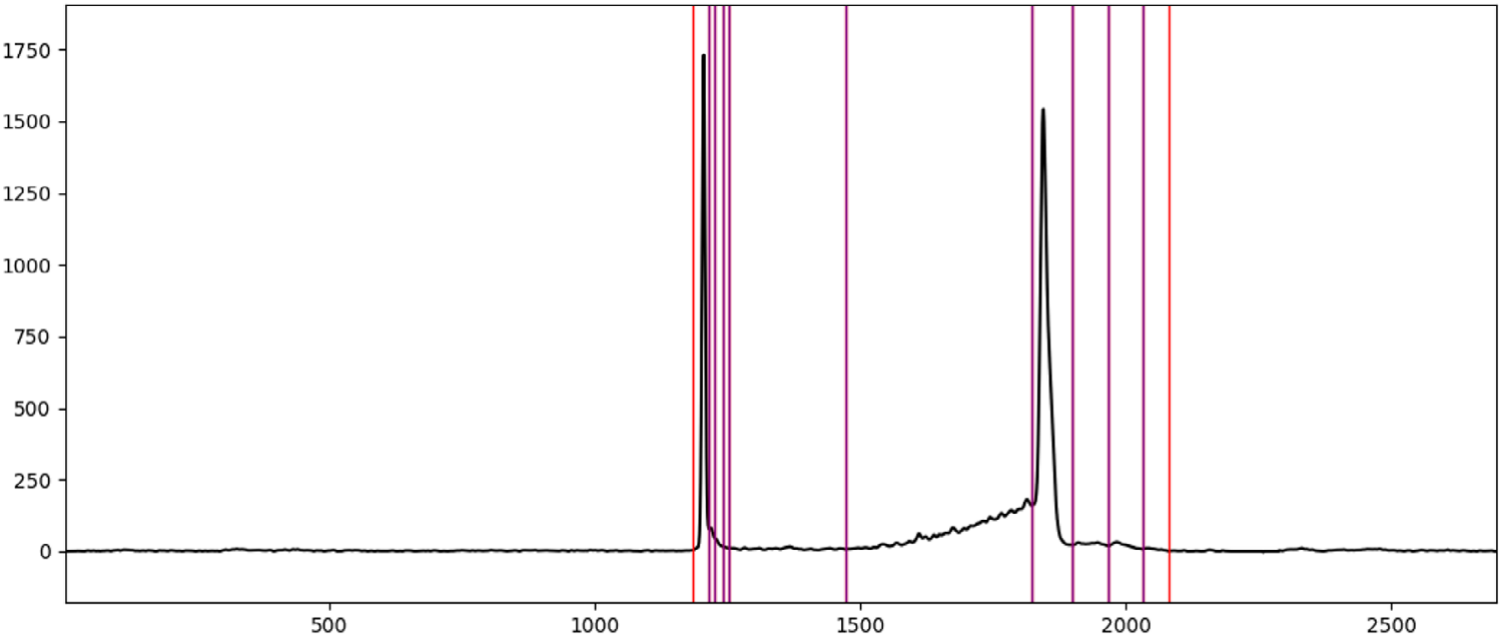
Electropherogram of an RNA degradation measurement. Red markers indicate the start or the end of a peak(‐group), whereas purple markers represent integration limits shared by two peaks within a peak group. The electropherogram has been integrated in a way that summarizes all the individual degradation product peaks into a single peak, which is located before the main peak of the intact RNA.

In addition, the analytical task determines whether a peak group should be integrated as a whole or whether its constituent peaks should be integrated. For example, when investigating charge variants of mAbs, it often makes sense to consider all basic and acidic impurities as a whole [7, 8, 9, 10]. When investigating mRNA purity, fragments often elute as a broad shoulder before the main peak. In this context, the sum of all impurities is decisive; the quantification of a specific species is of lesser interest (Figure 10).

In this collaborative study, we analyzed the data sets without regard to context to identify general rules applicable to all kinds of analyses. We are aware that this can be an artificial situation. The derived rules should be used in practice in conjunction with the relevant contextual knowledge.

#### 3.6.2 Peak Integration: Variance Component of the Total Error in CE

Sample pretreatment and integration are significant sources of error in LC [11, 12], which also applies to CE [13, 14]. Other significant sources of error include the stability of the EOF and associated surface phenomena [9, 15, 16].

These references show that the integration error becomes more relevant as analyte quantities decrease and areas become smaller, a phenomenon confirmed for CE in the following sections (see Section 3.7). For LC, it has furthermore been shown that the integration error becomes a less important source of error relative to sample preparation when the S/N value exceeds 100, assuming a good separation method [17].

### 3.7 Variability Using Manual Integration

Differences among the experts’ integrations demonstrate that manual integration is still subject to error. Given the current state of the art, manual integration is probably less error‐prone than software‐based integration for certain critical data sets (Tables 4 and 5), but it is not error‐free. The data generated in this work can be used to investigate this further by comparing the highest and lowest peak areas obtained from plausible integrations performed by different experts.

The following section examines the differences between the end and start times of four peaks from the com7‐type1‐sam4 run and the resulting areas as a first example. More detailed investigations on this topic will follow in future work. For each peak, our experts determined the boundaries based on their experience. We then calculated the ranges between the start and end of each peak for each expert and compared the relative differences in the ranges and the resulting areas.

**FIGURE 11:**
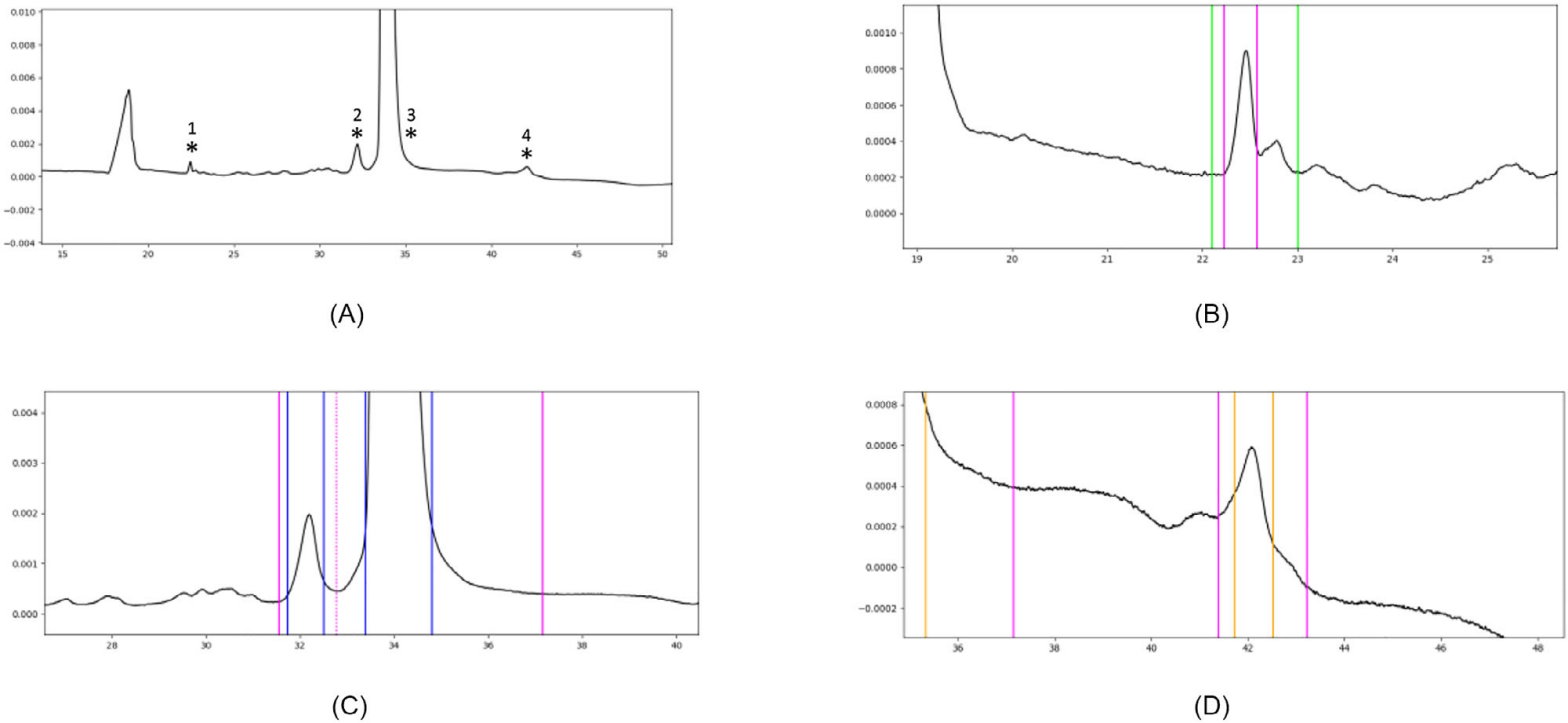
Varying approaches of our experts in integrating the four main peaks of the electropherogram com7‐type1‐sam4. The main peaks have been marked by an asterisk in (A). Integration limits from the same expert and, thus the same approach, are displayed in the same randomly assigned color, whereas integration limits from a different expert are displayed in a different color. (A) Raw electropherogram in zoomed state. (B) Peak 1 displayed with the broadest and narrowest expert limits. (C) Peaks 2 and 3 displayed with the broadest and narrowest expert limits. (D) Peak 4 displayed with the broadest and narrowest expert limits.

Figure 11 shows the largest and smallest peak areas determined by the experts. The resulting variability is shown in Table 3.

**TABLE 3:** Comparison of the variability in the integration limits set by our experts and the resulting variability of peak areas with regard to the different sized main peaks of electropherogram com7‐type1‐sam4.

Peak	Area from total (%)^a^	Relative difference between the widest and narrowest time spans determined by experts (%)^b^	Relative difference between the highest and lowest resulting area (%)^c^
1	0.29	61.6	72.2
2	1.48	73.4	52.6
3	97.7	211	5.94
4	0.56	131	115

^a^ Calculation: $\mathrm{Area}\;\mathrm{from}\;\mathrm{total}\;\left[\%\right]=100\cdot \frac{\mathrm{Are}a_{n}}{\mathrm{Are}a_{\mathrm{Total}}}$, where Area*_n_* is the area of the *n*th peak based on the reference integration limits. Area_Total_ is the sum of the *n* peak areas based on the reference limits.

^b^ Calculation: $\mathrm{Time}\;\mathrm{span}\;\mathrm{difference}\;\left[\%\right]=100\cdot \frac{\left(x_{b,\;\mathrm{end}}-x_{b,\;\mathrm{start}}\right)-\left(x_{n,\mathrm{end}}-x_{n,\mathrm{start}}\right)}{\left(x_{n,\mathrm{end}}-x_{n,\mathrm{start}}\right)}$, where *x_b_*_,end_ is the time value of the integration limit that marks the end of the peak, which is defined by the broadest time span as determined by our experts. *x_b_*_,start_ is the time value of the integration limit that marks the start of the peak, which is defined by the broadest time span as determined by our experts. *x_n_*_,end_ is the time value of the integration limit that marks the end of the peak, which is defined by the narrowest time span as determined by our experts. *x_n_*_,start_ is the time value of the integration limit that marks the start of the peak, which is defined by the narrowest time span as determined by our experts.

^c^ Calculation: $\mathrm{Area}\;\mathrm{difference}\;\left[\%\right]=100\cdot \frac{\mathrm{Are}a_{\mathrm{High}}-\mathrm{Are}a_{\mathrm{Low}}}{\mathrm{Are}a_{\mathrm{Low}}}$, where Area_High_ is the highest area determined by our experts. Area_Low_ is the lowest area determined by our experts.

Besides the zooming level, the size of the peaks appears to have a significant influence on the error in manual integration, as these initial investigations suggest (see Section 3.6.2). Small peaks appear to be particularly sensitive to different integration limits, whereas the areas of larger peaks appear to be less affected by setting different peak limits.

The comparison of precision in calculating peak areas and peak size indicates a numerical relationship: Precision is proportional to the inverse of the square of the peak size:

$$\mathrm{Precision}∼\frac{1}{\sqrt{A}}$$

This finding is consistent with Dyson's earlier comments on integration errors [18].

The example also illustrates that the peak shape appears to have a notable influence on the variability of our experts’ limits. The greater the asymmetry of a peak, the greater the divergence in experts’ estimates, especially when the peak shows significant fronting or tailing.

**FIGURE 12:**
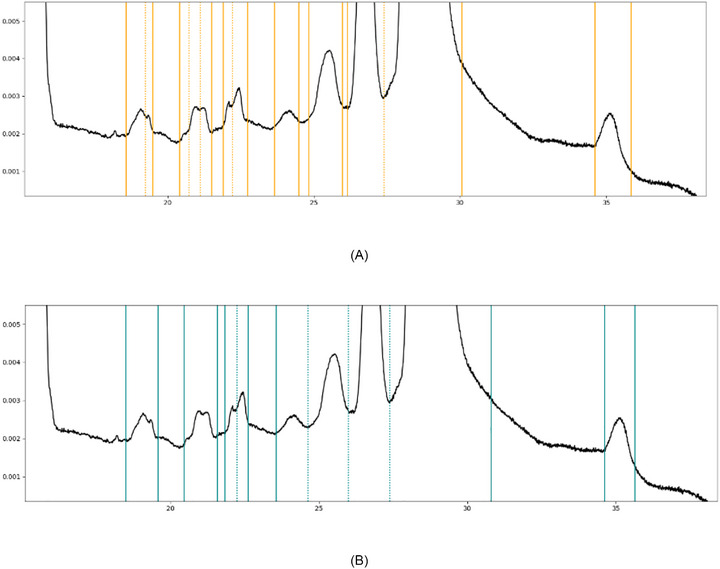
Example of an electropherogram being integrated twice by the same expert. When integrating the duplicate of the electropherogram from (A) at a later stage of the series, the expert chose to make slight changes to decisions regarding baseline separations, shoulder inclusion, and the general placement of the integration limits (B).

Interestingly, variability in manual integration can be observed not only among different experts but also among the same expert when integrating the same electropherogram at a later stage (Figure 12). This observation was made possible by adding duplicates to the subset of electropherograms each expert was asked to integrate at the beginning of the project (see Section 2.3). The original electropherograms and their duplicates were both part of a larger series of similar‐looking electropherograms within the subset. However, they did not share the same name, nor did they follow one another chronologically, making it unlikely for the expert to identify the duplicates while working through the series.

To minimize manual integration errors, it is therefore advisable to select the correct zoom levels and follow a set of rules (Section 3.4, Supporting Information).

### 3.8 Plans for Further Investigations Using Reference Data Set No. 1

The next critical step is to compare the performance of state‐of‐the‐art commercial software for automatic peak integration with the results obtained from reference data set 1. A comprehensive evaluation of this topic, including Chromeleon, Empower, and other instrument‐specific softwares, will be presented in future work.

**FIGURE 13:**
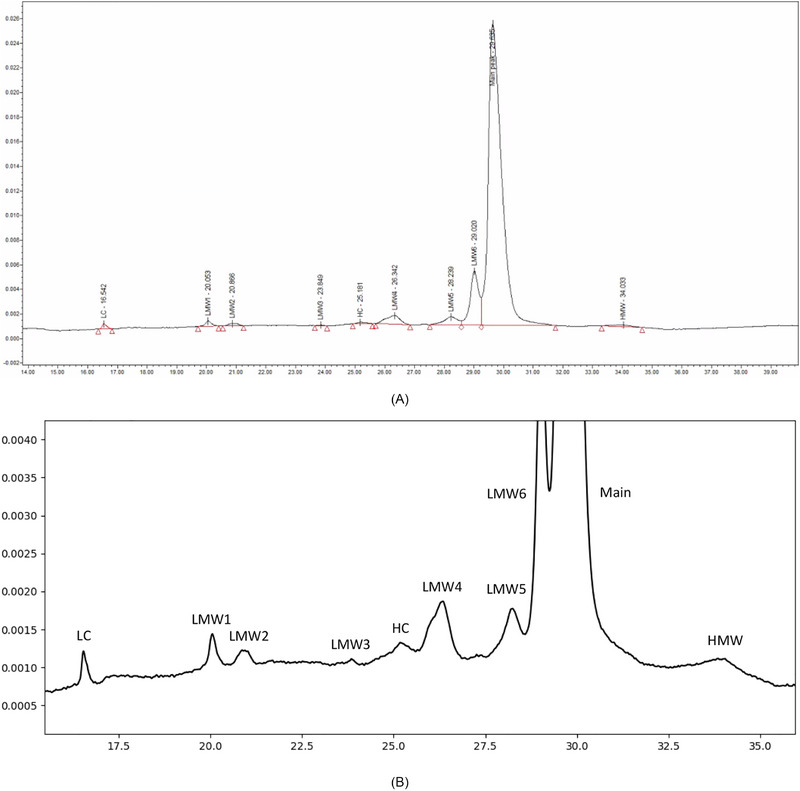
A representative electropherogram integrated automatically by Empower. The electropherogram is displayed at full‐scale (unzoomed), showing Empower's set integration limits (A), and with enhanced magnification (zoomed‐in), without integration limits, to provide a detailed view of the baseline and minor peaks (B).

As a preliminary evaluation, we analyzed a data set of 21 runs in terms of their precision in automatic integration with established software or manual integration with our reference integration limits. An example of this is shown in Figure 13. These electropherograms were first integrated using an earlier version of Empower and then integrated by our team using “Manual Integrator” (see Section 2). In automatic integration mode, we obtained the integration limits and corresponding peak areas using Empower's trapezoidal integration function. We then re‐integrated the peaks using the same trapezoidal function, applying the reference integration limits previously agreed upon by our experts. The results are summarized in Table 4. Although Empower's automatic integration performed reasonably well, the expert‐based manual integration provided better overall results.

**TABLE 4:** Relative standard deviations (RSD%) of the calculated peak areas from the 21 electropherograms of the com6‐type1 series based on the integration limits automatically set by the software Empower and the reference integration limits consolidated by our experts.

	Peak area RSD%									
	LC	LMW1^a^	LMW2^a^	LMW3^a^	HC^a^	LMW4	LMW5	LMW6	Main^a^	HMW
Empower (earlier version)	15.43	8.13	25.56	28.60	24.82	11.96	23.04	3.20	1.36	43.80
Manual Integration^b^	12.40	5.75	10.08	13.60	17.76	9.94	19.16	3.19	1.01	34.17

Abbreviation: LC, liquid chromatography.

^a^ The peaks where the values significantly differ are marked with an asterisk.

^b^ The manual integration is based on our reference integration limits.

Some of the observed differences in the standard deviations of the corresponding estimated peak areas within the series were statistically significant at a level of *α* = 0.10 using a Fisher's *F*‐test. Here, *α* denotes the probability of a type I error, that is, detecting a difference in variability that is due solely to random variation. We selected a relatively liberal significance level of 10% in order to reduce the probability of a type II (*β*) error, that is, failing to detect an existing difference in variability. Although manual integration resulted in lower variability across all peaks examined, the difference was statistically significant in the pairwise comparisons for five peaks. These findings indicate that current commercial programs perform well but that further improvement is possible.

### 3.9 Testing the Derived Rules on Other Data Sets

**FIGURE 14:**
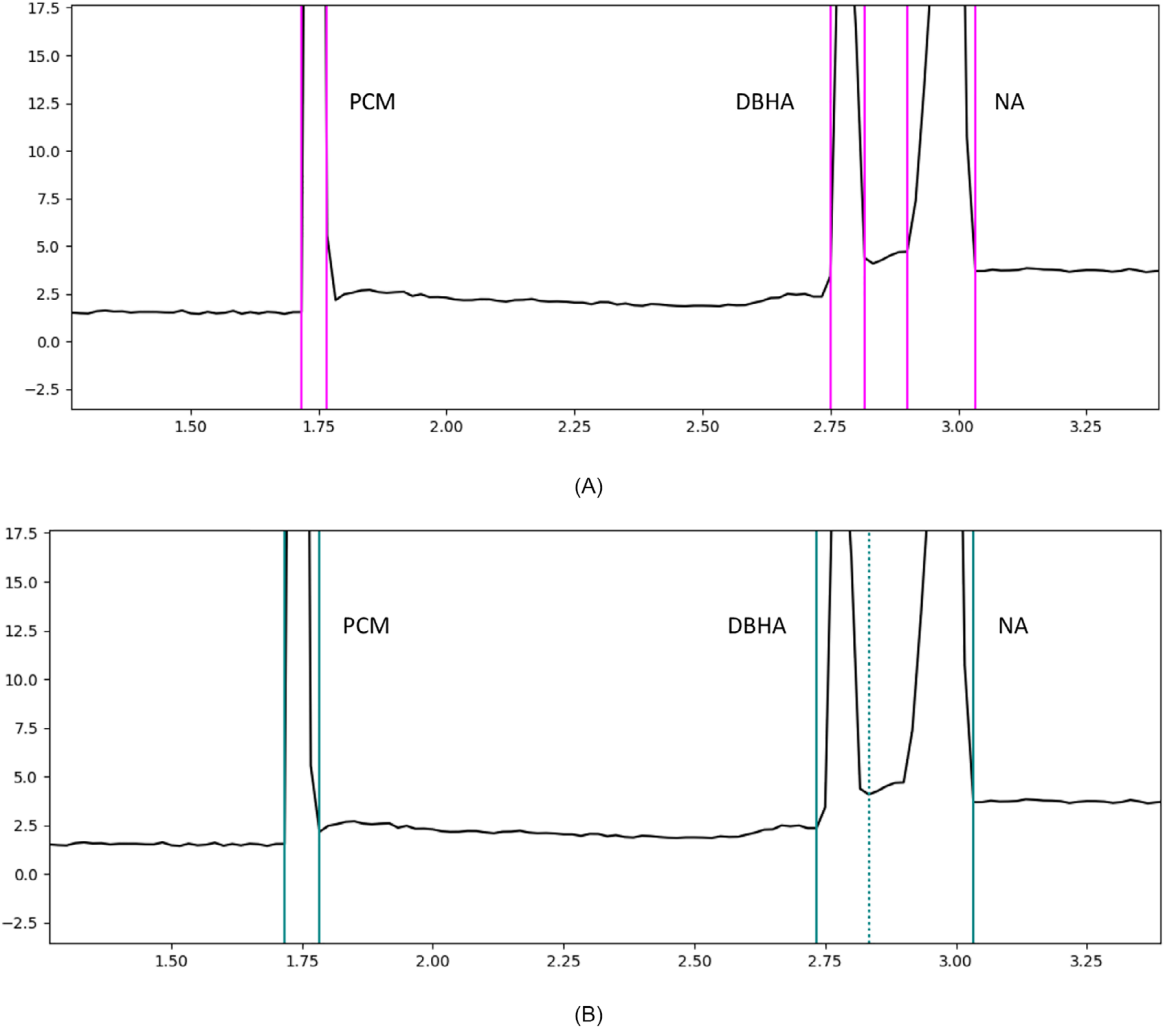
Electropherogram of one of the ten injections of the system suitability test. The electropherogram was integrated automatically by the DataApex software Clarity (A) and manually according to the rules of this publication (B). DBHA, 2,4‐dihydroxybenzoic acid; NA, nicotinic acid; PCM, paracetamol.

A system suitability test mixture (paracetamol [PCM], 2,4‐dihydroxybenzoic acid [DBHA], and nicotinic acid [NA]) was analyzed 10 times under constant conditions using the PrinCE instrument. Automatic integration was performed using the included Clarity software from DataApex. We then manually integrated the peaks according to the postulated rules from our collaboration (see Section 3.3). The relative standard deviation (RSD%) for each peak area was significantly higher using the Clarity software (*α* = 10%). Furthermore, this simple test mixture is relatively straightforward to integrate, particularly compared with large biomolecules, which frequently yield complex peak profiles that are more difficult to integrate (Figure 14). The results can be found in Table 5.

**TABLE 5:** Relative standard deviations (RSD%) of the calculated peak areas based on the integration limits automatically set by PrinCE's included clarity software from DataApex and the integration limits manually set in accordance with the rules of this publication.

	Peak area RSD%		
	Paracetamol (PCM)	2,4‐Dihydroxybenzoic acid (DBHA)	Nicotinic acid (NA)
Clarity	15.33	16.65	23.55
Manual integration	5.89	8.75	6.08

## 4 Conclusion and Outlook

A consolidated reference data set for peak integration in CE is presented here for the first time. It consists of 227 relevant and challenging electropherograms. In total, more than 1700 data files are available, each containing either the electropherograms in original form, the corresponding integration limits after individual integration by various experts, or the finally agreed reference integration limits. This data set enables an objective comparison of different integration approaches, including evaluations of the performance of different software products and versions, as well as comparisons of automatic and manual integration. It can also be used to develop improved peak‐integration algorithms and to provide training materials for analysts, including the development of objective performance indicators. We anticipate that further studies based on this consolidated data set will demonstrate its value for improving both automatic and manual integration.

Knowledge of the origin and composition of both the analytes and the sample matrix is essential for meaningful integration in routine practice. In this work, we generally analyzed the data sets without using this contextual information to derive general, consistent integration rules. These rules are intended to be applied in practice alongside relevant contextual knowledge.

We recommend collecting as much contextual information as possible during method development and validation to thoroughly identify potential integration challenges and define detailed integration instructions and rules. These studies should include a series of measurements using different samples over a range of concentrations that are representative of the peaks and relative abundances expected in routine use. Higher sample concentrations can help confirm the presence of peaks and aid in distinguishing true peaks from noise at lower concentrations. Forced degradation studies are particularly valuable for generating samples that represent the range of peaks and concentrations that integration instructions should address.

It is reasonable to anticipate competition among CDS vendors to enable fully automated integration through improved algorithms. We are optimistic that future improvements in CDS algorithms will make automatic integration feasible for essentially all CE data sets. The list of integration parameters described in reference [1] is expected to be completed. We are also confident that the present work will contribute to the development of practical integration guidelines specifically for CE. At the current stage, whereas automatic integration is still being optimized, discussions among experts have highlighted several challenges associated with manual integration, including the treatment of fronting and tailing peaks, correction of baseline irregularities, and the choice of an appropriate zoom level for precise peak delineation. The results of these discussions are also useful for developing practical guidelines and SOPs for manual integration. These SOPs can be clearly illustrated using data from the reference data set.

The consolidated reference data sets will be used to evaluate algorithmic and AI strategies under development to enhance peak integration. We plan to apply these reference data sets to assess the performance of established commercial software packages and objectively rate their ability to achieve automatic integration. We will also investigate the advantages and disadvantages of different smoothing algorithms, as well as potential strategies to improve precision, such as using peak height instead of peak area, as described by Dyson [19], or integrating only a defined portion of each peak to reduce the influence of noise in the peak flanks. Preliminary investigations already indicate that errors in setting integration limits have a smaller impact on large peaks than on small peaks. In particular, substantial precision issues can arise when small peaks are not integrated correctly.

A further challenge concerns small peaks and the question of when a feature should be regarded as a true peak. Electropherograms can contain many peaks of widely varying sizes, including very small peaks that are only slightly above the baseline noise. In addition to application‐specific decision rules, robust signal‐based criteria are needed to define appropriate peak baselines. Such criteria could, for example, be derived from the S/N ratio definitions used for detection limits in the pharmacopoeias. The selection of peak baseline functions will also be examined in future work, as baseline choice strongly influences the calculated peak area, particularly for small peaks.

AI has the potential to become a valuable tool for supporting automatic peak integration in the future. In this project, we will investigate AI‐based strategies and train AI models using the consolidated data sets. The performance of AI‐driven integration will be carefully compared with that of conventional algorithmic approaches, with particular attention to regulatory considerations for AI use.

If AI proves effective in fully automating peak integration, it could be combined with other AI applications to systematize APs and data management, for example, by identifying optimal method parameters through comparison of current and historical data [20].

To facilitate data‐intensive training of AI models, we plan to expand the available data by collecting additional experimental data from collaborators and, if needed, by generating simulated data derived from the reference data set. In addition to consolidating data from a larger number of samples, we are particularly interested in data sets that include blank injection runs, because we expect AI to have significant potential for developing and applying baseline models. In practice, baselines often exhibit apparently random changes over time, which makes them difficult to predict. We therefore aim to construct baseline models from multiple blank runs that can reliably predict the baseline course in analytical injections, as we observe limitations in conventional algorithms in this area. Both improved algorithmic approaches and AI‐based methods appear promising in this regard. It is desirable to have generally valid rules for all applications or data types from CE. If this is not readily possible, special rules for specific applications could be another solution.

**FIGURE 15:**
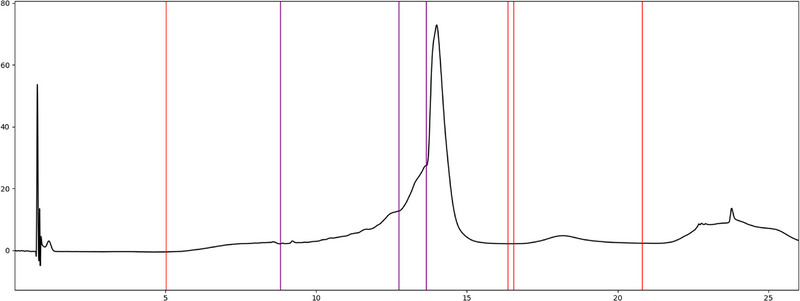
Chromatogram of an ion pair measurement of degraded mRNA in lipid nanoparticles. The chromatogram has been manually integrated based on the integration rules of this publication.

We are confident that this research will yield practical, experimentally grounded integration guidelines specifically for CE and that the underlying concept will be transferable to other techniques such as LC (Figure 15). Rules developed for CE are likely to be at least partially applicable to LC, and conversely, experience from LC integration can inform CE practice [4]. In parallel, consolidated reference data sets can be compiled by the LC community in a manner similar to that described here, establishing a standard, data‐driven foundation for objective comparison of integration strategies. We believe the time has come to automate the integration of electropherograms fully. Achieving this goal will require sustained collaboration among CE users, instrument and CDS vendors, and other stakeholders, but with shared reference data and transparent rules, robust, fully automated integration is now a realistic and attainable objective.

The data set published here consists of carefully selected and curated electropherograms, which were then evaluated and discussed in depth by a large group of experts, who agreed on the specified peak limits. However, it is not yet possible to verify their accuracy. The data sets presented here can be supplemented and improved by additional data sets that enable statistical comparison. Data sets in which the same or a similar sample has been measured multiple times, for example, in calibration experiments, stability tests, or process analytics, are useful here. The data set may also be supplemented by electropherograms covering additional properties, such as specific baseline characteristics. All data sets that contain additional information are certainly valuable.

Everyone is welcome to use the reference data for their own purposes, including utilizing only parts of the data. This could mean using only a few of the 227 electropherograms in total or using only a few of the given integration limits within a respective electropherogram, for example, due to disagreement with certain limits. However, if a data file containing integration limits is altered in any way, for example, by excluding or modifying some of the given reference limits, the altered file must be clearly labeled such, including detailed information about the change.

At present, it is still difficult to achieve objectively perfect integration with sufficient accuracy and precision when analyzing complex samples such as biopharmaceuticals. Every analyst has their own opinion about the best possible integration approach. However, by discussing and exchanging ideas and agreeing on common approaches, we are moving closer to objectivity. Therefore, we consider the published reference limits and integration rules to be preliminary and open to future changes. We welcome further discussion on this published data set no. 1, which will lead to an improved reference data set no. 2 in the future.

## Conflicts of Interest

The authors declare no conflicts of interest.

## Supporting information




**Supporting File 1**: elps70077‐sup‐0001‐SupMat.zip.

**Supporting File 2**: elps70077‐sup‐0002‐RawElectropherograms.zip.

**Supporting File 3**: elps70077‐sup‐0003‐SupMat.zip.

**Supporting File 4**: elps70077‐sup‐0004‐ReferenceDataSetNo.1.xlsx.

**Supporting File 5**: elps70077‐sup‐0005‐SupMat.pdf.

## Data Availability

The data that support the findings of this study are openly available in TU Braunschweig Cloud Server at https://cloud.tu‐braunschweig.de/s/4rNBxzdL7snS3Te.
