# Supplementary material for: Improvement of Peak Integration in Capillary Electrophoresis: Reference Data Set No. 1
Source: Electrophoresis. 2026 Mar 11;47(4):276–94. doi: 10.1002/elps.70077 (PMC13084974; doi:10.1002/elps.70077)
Supplement: Supplementary file 5 — Supporting File 5: elps70077‐sup‐0005‐SupMat.pdf. [file ELPS-47--s003.pdf]

Integration Manual

Part of the scientific article "Improvement of peak integration in Capillary Electrophoresis: Reference Data Set No. 1" by

Marlon Krompholz[1], Timothy Blanc[2], Huixin Lu[3], Patricia Christensen[6], Frédéric Ginot[7], Gábor Járvás[8], Trang D. Nguyen[9], Ashley Prout[6], Timothy Riehlman[10], Brian Wei[11], Andrei Hutanu[12], Steffen Kiessig[12], Knut Baumann[1], Cari E. Sänger – van de Griend[1,4,5], Hermann Wätzig[1]

Submitted to Electrophoresis in 2025.

1 Appropriate Display in Three Steps

This manual describes the integration approach used in the Intercompany Collaboration (ref) and serves as a guide for future work with the reference data set. It also provides guidance for reviewers to assess whether integrations have been performed correctly.

The analyst determines the integration limits in electropherograms by successively applying different methods. Viewing electropherograms at various degrees of magnification (zoom levels) is a critical step in determining the appropriate integration limits (peak start and peak end) of electropherograms. When working at these various zoom levels, some Basic Rules (see below) should be followed.

Level 1: Full-Scale (No zoom)

View the electropherogram in full-scale first. Set markers at the start and end of every clearly recognizable peak in the electropherogram.

Level 2: Moderate zoom (e.g. y-scale x 15, x-scale x 3)

Zoom in to obtain a more detailed view of the peak. Adjust the integration limits set at Level 1 as needed to improve their placement. If additional peaks become visible at this zoom level, set start and end markers for each new peak.

Level 3: Highly detailed zoom (e.g. y-scale x 100, x-scale 6)

Zoom-in further to achieve a very detailed view of the peaks. Adjust integration limits according to the Specific Rules (see below).

See Importance of the zoom

Moving progressively from low detail (Level 1) to very high detail (Level 3) helps the analyst maintain perspective and context when defining peak boundaries. At very high zoom, it becomes difficult to distinguish potential peaks from surrounding noise. Therefore, it is not recommended to start directly at Level 3; instead, the zoom level should be increased stepwise to preserve an overall view of the electropherogram.

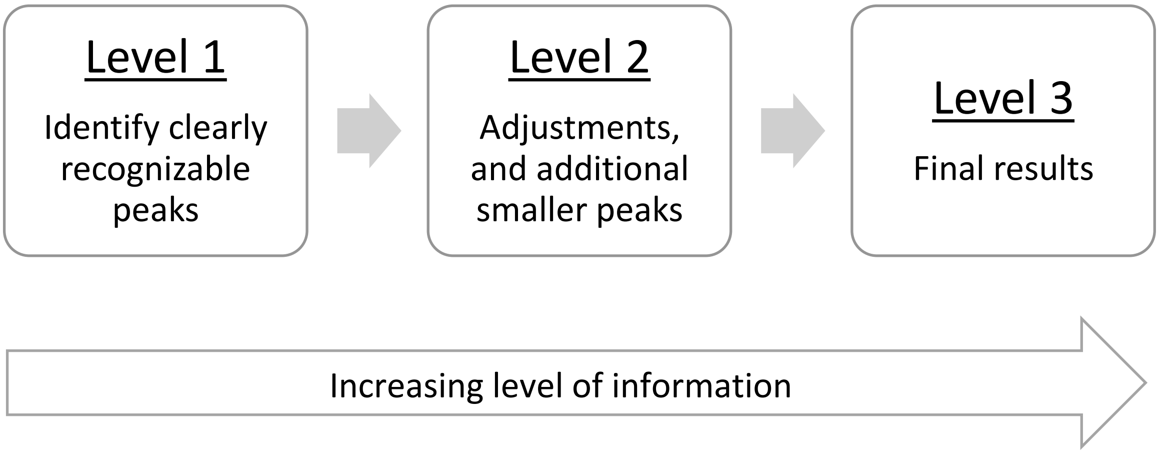

Rules for Peak and Peak Limit Determinations

2 Basic Rules

1. When peaks are obvious and definitively emerge from the underlying baseline they should be marked at the beginning and end of this emergence. Whenever there is any uncertainty as to whether an area represents a peak or part of the baseline, the area in question will be considered as a peak if it meets either of the following criteria:
    - It exceeds a signal-to-noise ratio of 3 relative to the surrounding noise (*See Example 2.1*) or
    - The peak is present at higher levels in related samples (e.g., a later stability timepoint) from the same series or not present in blank injections associated with the sample analysis (*See Example 2.2*).
2. When two peaks merge because of an insufficient baseline separation, they always share a marker. The shared marker represents both the end of the first peak and the beginning of the following peak (*See Example 2.3*).
3. It is essential to always consider the baseline noise. If two peaks are unresolved, but the section between them shows a noise level consistent with the rest of the electropherogram, the two peaks will not be considered a peak group and therefore will not share a marker (*See Example 2.4*).
4. If a section of the electropherogram shows an obvious noise pattern but clearly lays above the expected baseline, this section will be considered as a peak (*See Example 2.5*).
5. A shoulder is an unresolved peak on the leading or trailing edge of a larger peak. To be treated as a shoulder, the signal must show either
    - a local minimum relative to the main peak, or
    - a distinct inflection region on the main peak where the local slope is approximately zero.

   If no such local minimum or zero-slope inflection is present, or if this feature could reasonably be interpreted as baseline noise, the region is not considered a shoulder unless comparison with other injections in the same series clearly confirms a reproducible feature at the same migration position (*See Example 2.6*).

   Shoulders do not need to meet the requirement of exceeding a signal to noise ratio of 3 to be considered.
6. Negative peaks are taken into account and will therefore be marked in compliance with the other basic rules (*See Example 2.7*).
7. Peaks that can be clearly identified as system peaks and are therefore not related to the sample will not be included in the integration. The presence of system peaks can be confirmed by blank injections without analyte (*See Example 2.8*).

General

1. Does the peak emerge from a completely flat region of the baseline (No slope)?
   - Yes → Beginning/end of slope.
     See Example 3.1
   - No → Apply any of the following rules.

2. Is there a peak immediately before the considered peak (unresolved peaks)?
   - Yes → Valley (Local minimum) between the peaks.
     See Example 3.2
     If the valley is overlaid by noise, apply Rule 3.9 or Rule 3.10.
   - No → Is there a local minimum immediately before the main increase?
     - Yes → Local minimum.
       See Example 3.3
     - No → Is there an inflection point immediately before the main increase?
       - Yes → Inflection point.
         See Example 3.4
       - No → Point where the increase significantly changes its rate.
         See Example 3.5

3. Is there a peak immediately after the considered peak (unresolved peaks)?
   - Yes → Valley (Local minimum) between the peaks.
     See Example 3.2
     If the valley is overlaid by noise, apply Rule 3.9 or Rule 3.10.
   - No → Is there local minimum immediately after the main decrease?
     - Yes → Local minimum.
       See Example 3.6
     - No → Is there an inflection point immediately after the main decrease?
       - Yes → Inflection point.
         See Example 3.7
       - No → Point where the decrease significantly changes its rate.
         See Example 3.8

4. Does the considered peak include shoulders above the baseline?

   Yes → Is there a valley (local minimum) between the considered peak and its shoulder?

   - Yes → Valley (Local minimum).
     See Example 3.9
   - No → Is there an inflection point between the peaks?
     - Yes → Inflection point.
       See Example 3.10
     - No → Point where the slope of the considered peak significantly changes its rate.
       See Example 3.11

   No → Do not divide the peak into two peaks.

5. Does the considered peak include fronting?

   Yes → Point where the front exceeds the expected baseline course.
   See Example 3.12

   Please note:
   The start of a front-side shoulder can be difficult to define because it gradually rises from the baseline. To set the start limit, first examine the preceding baseline noise. Place the start marker at the point where the signal is consistently elevated above the baseline noise for the first time.
   Apply Rule 3.2 to this peak.

6. Does the considered peak include tailing?

   Yes → Point where the tail falls below the expected baseline course.
   See Example 3.13

   Please note:
   The end of a tail can be difficult to define because it gradually returns to the baseline. To set the end limit, first examine the baseline noise that follows the peak. Place the end marker at the point where the signal first returns to and stays within the expected baseline noise range.
   Apply Rule 3.2 to this peak.

Purity Analysis

7. Does there appear to be a series of unresolved peaks/shoulders on the front side of a peak?
   See Example 3.14

   Yes → Point where the front-like area exceeds the expected baseline course.
   (Apply Rule 3.5)
   See Example 3.15

   + Point of transition between distinct subunits of the front-like area.
   A subunit is always created when its area deviates from the expected course of the previous area.
   (Apply Rule 3.2)
   See Example 3.16

   + Point of transition from the front-like area to the main peak.
   (Apply Rule 3.2)
   See Example 3.17

8. Does there appear to be a series of unresolved peaks/shoulders on the backside of a peak?
   See Example 3.18

   Yes → Point of transition from the main peak to the tail-like area.
   (Apply Rule 3.2)
   See Example 3.19

   + Point of transition between distinct subunits of the tail-like area.
   A subunit is always created when its area deviates from the expected course of the previous area.
   (Apply Rule 3.2)
   See Example 3.20

   + Point where the tail-like area falls below the expected baseline course.
   (Apply Rule 3.6)
   See Example 3.21

Overlaid valleys

9. Is there a noise peak at the minimum of a valley?

   Yes → At the end of the noise flank that extends further downwards.
   See Example 3.22
   If both flanks end at the same height, place the integration limit on top of the noise peak.

10. Is there a relatively flat section between two unresolved peaks? Does the valley resemble a bridge between the peaks?

Yes → In the middle of the bridge.
See Example 3.23

Negative peaks

11. Is there a negative peak?

Yes → Invert the rules for positive peaks.
See Example 3.24

12. Does a negative peak directly transition into a positive peak (vice versa)?

Yes → Inflection point becomes the shared marker between the two peaks.
See Example 3.25

Examples

The following examples contain electropherograms with integration limits that correspond to the relevant rule. The integration limits are marked in red or purple. Red markers indicate the start or the end of a peak(-group), while purple markers represent integration limits shared by two peaks within a peak group.

1 Importance of the zoom (Back to Page 1)

Example workflows through different zoom levels

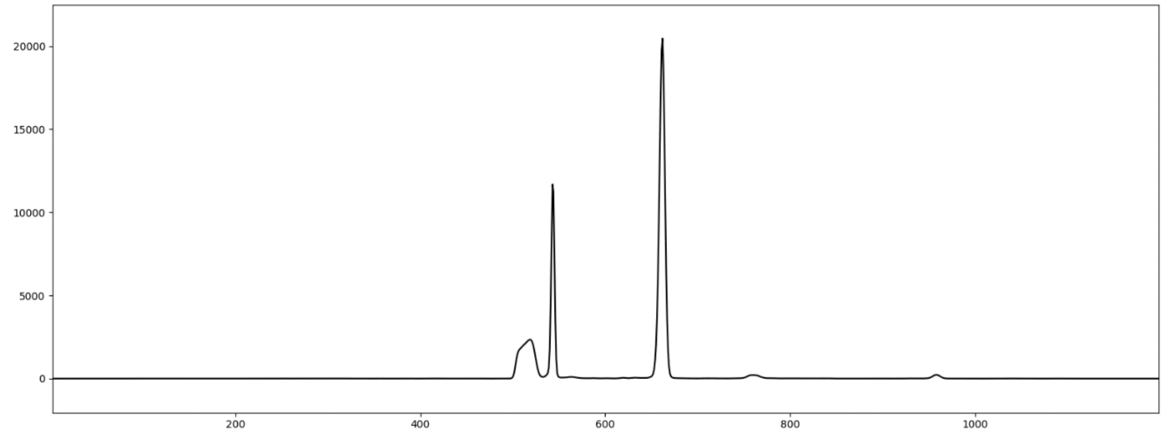

Figure 1: Electropherogram in unzoomed state (Level 1).

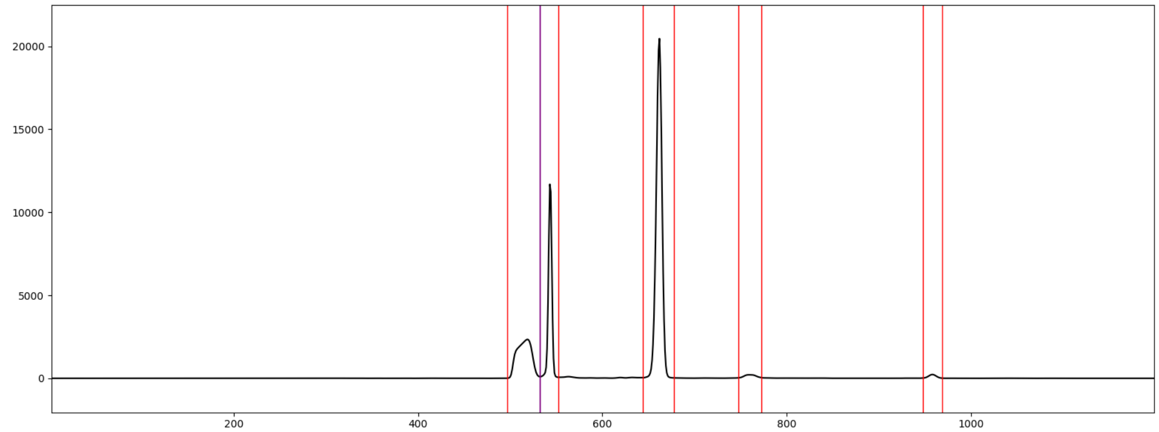

Figure 2: Marker generation in unzoomed state (Level 1).

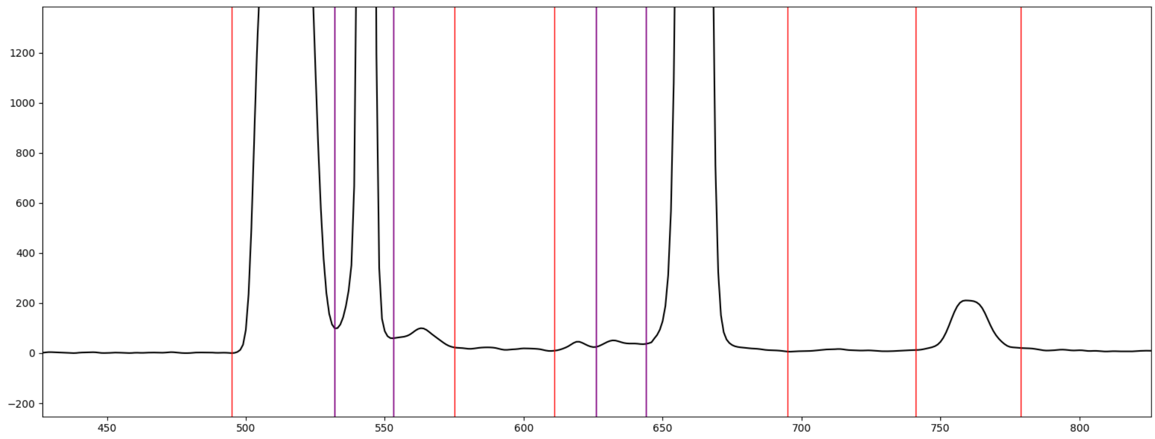

Figure 3: Marker adjustment and further generation in a moderate zoomed state (Level 2).

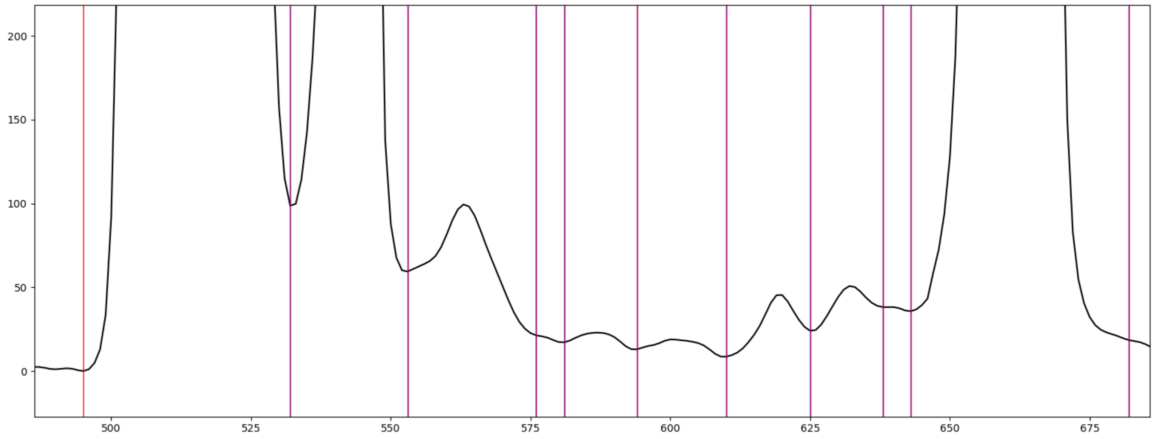

Figure 4: Marker adjustment and further generation in a highly detailed zoom state (Level 3).

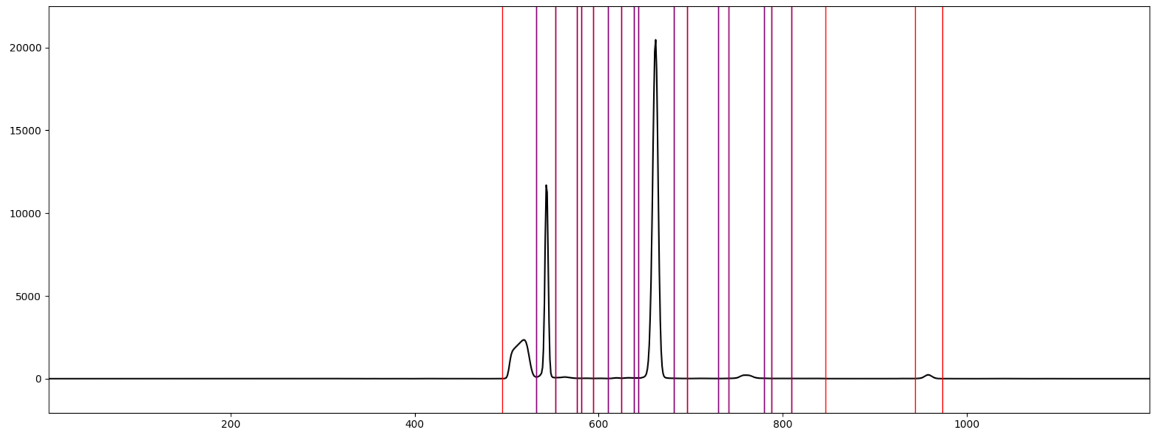

Figure 5: Final result in a full-scale view.

2 Basic Rules - Examples

Example 2.1 (Back to Basic Rules)

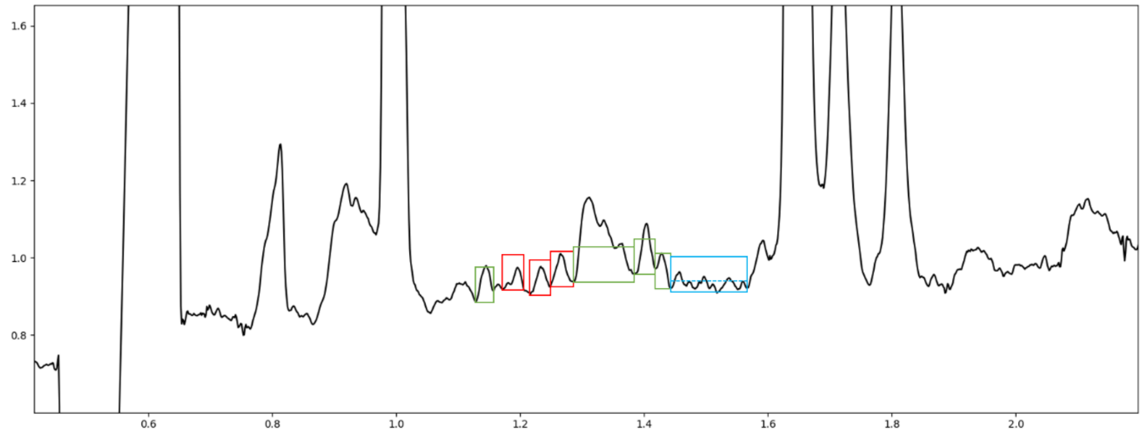

Figure 6: Illustration of small peaks relative to a signal-to-noise (S/N) ratio of 3. The blue box has a height three times the average baseline fluctuation (blue dotted line) in a nearby noise region. Boxes of the same height are placed over questionable peaks, with the bottom edge aligned to the lowest point of each peak. Peaks that do not reach the top of their box are shown in red; peaks that reach or exceed the box height are shown in green. Shoulders are treated as part of their main peak and are counted as peaks if the associated main peak meets S/N ≥ 3.

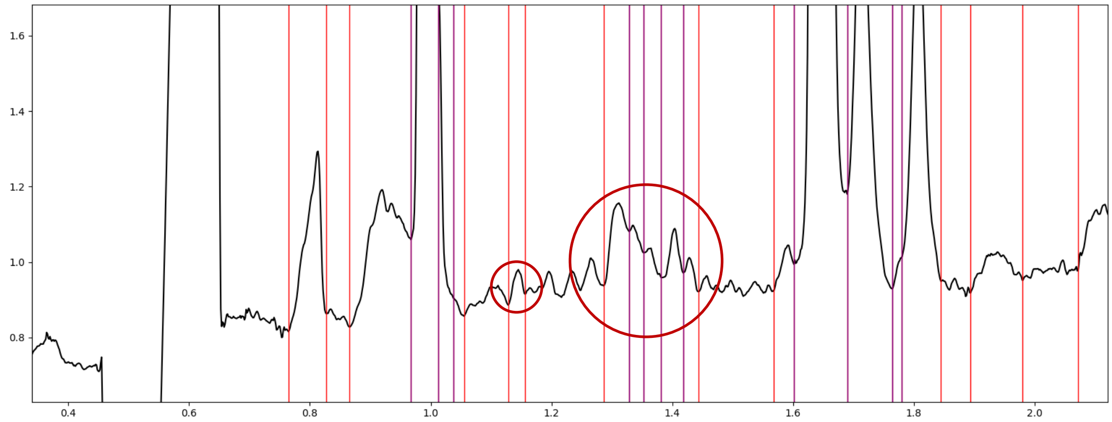

Figure 7: Integration limits of small peaks, that exceeded a signal-to-noise ratio of 3 relative to the surrounding noise.

Example 2.2 (Back to Basic Rules)

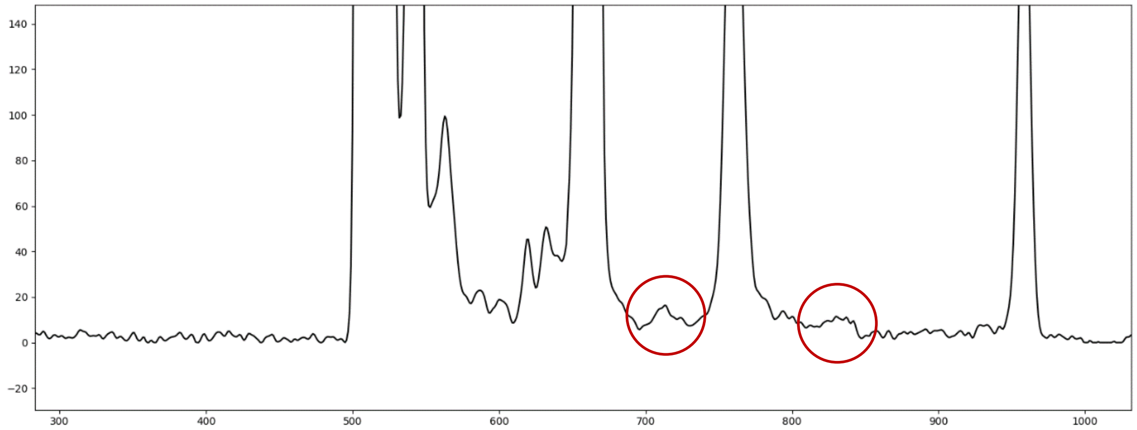

Figure 8: Electropherogram with areas that are difficult to classify as peaks or baseline movement without further information.

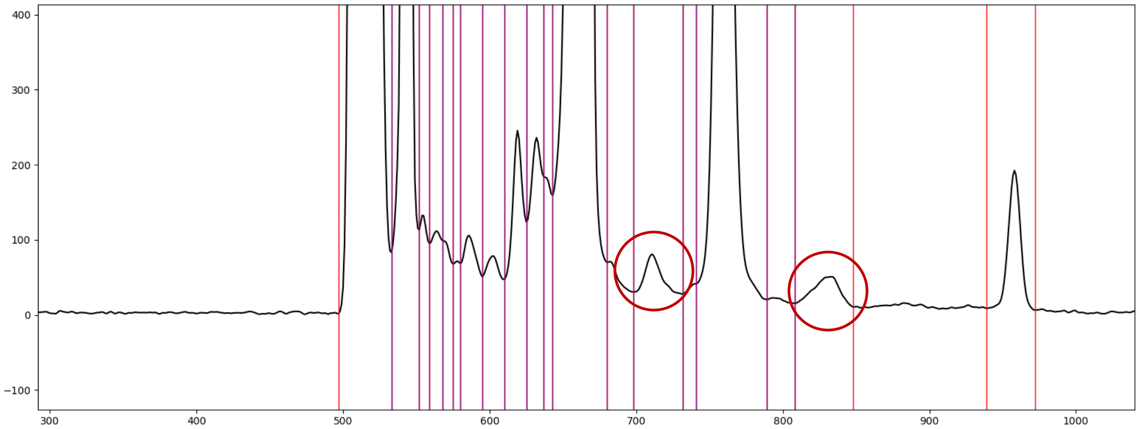

Figure 9: Electropherogram from the same series as Figure 8. In this example, the regions in question from Figure 8 can be clearly classified as peaks.

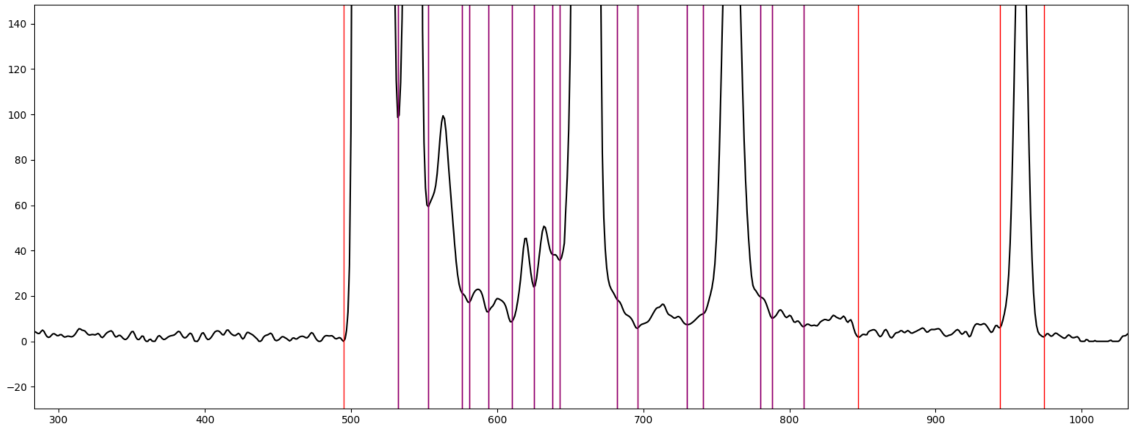

Figure 10: Final integration limits in the electropherogram from Figure 8 after comparison with the electropherogram from Figure 9.

Example 2.3 (Back to Basic Rules)

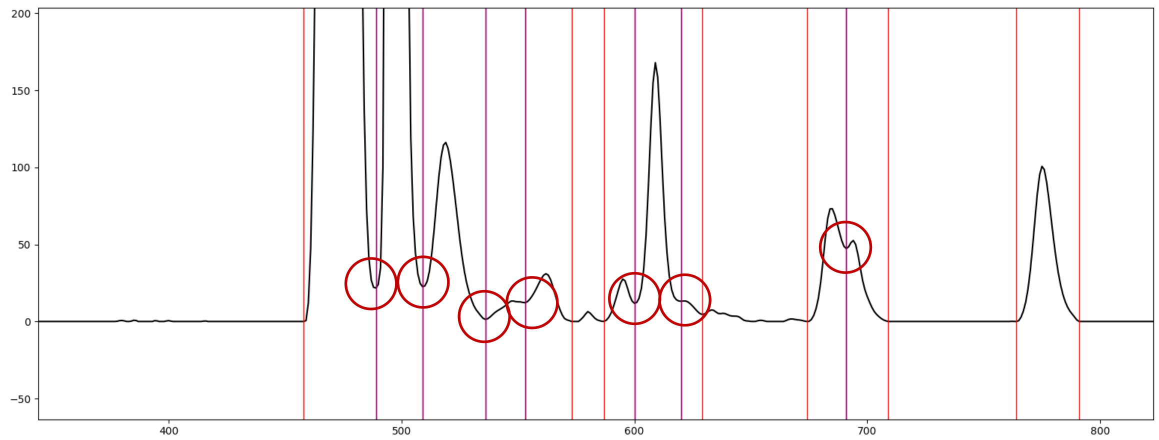

Figure 11: Unresolved peaks with shared integration limits. The shared integration limits are displayed in purple.

Example 2.4 (Back to Basic Rules)

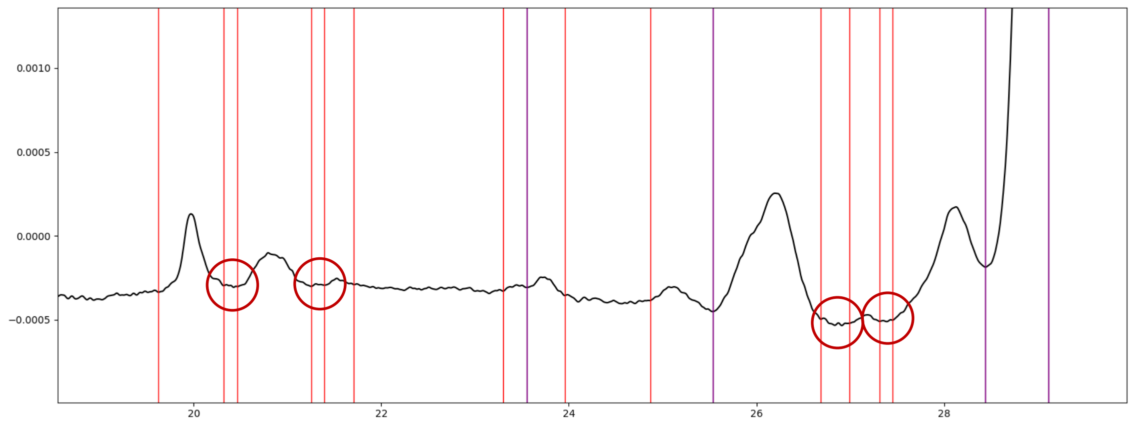

Figure 12: Peaks that seem to merge but are separated by a noise. Those peaks will not share an integration limit.

Example 2.5 (Back to Basic Rules)

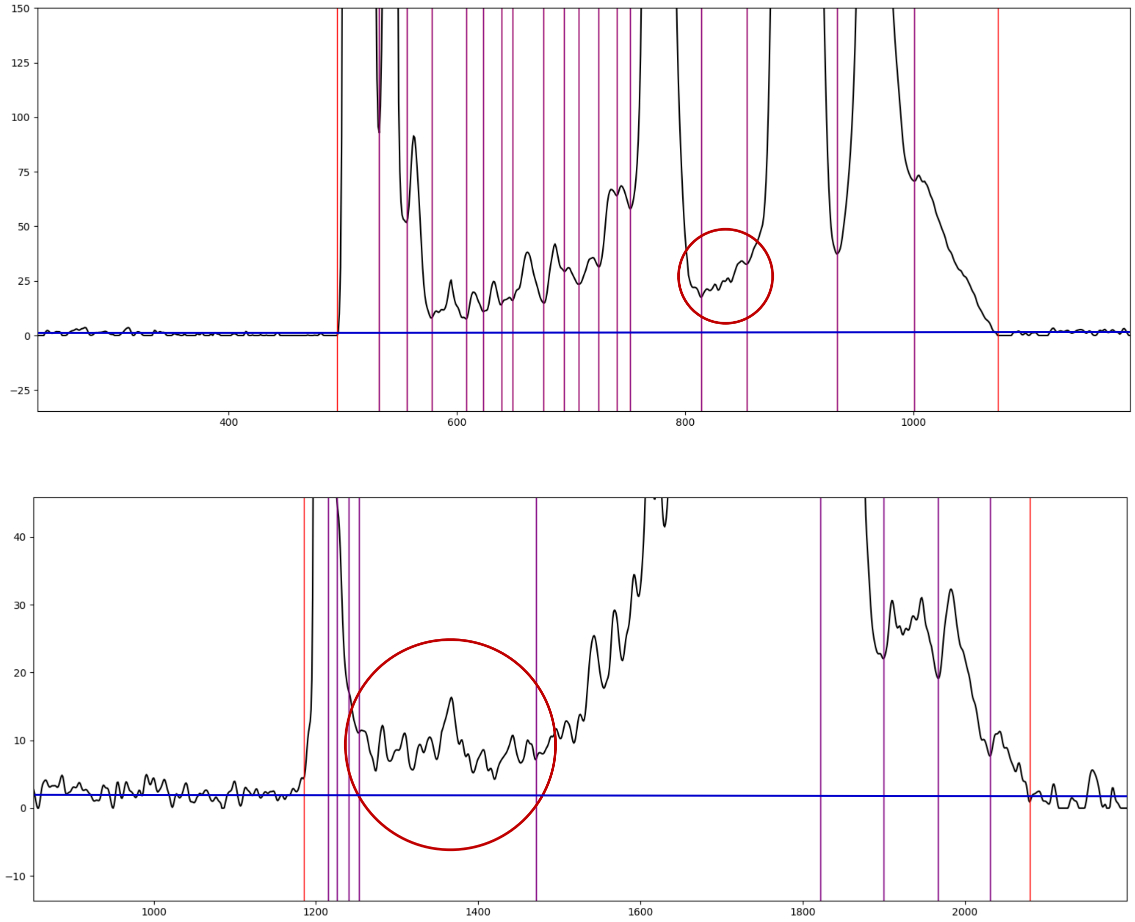

Figure 13: Two example electropherograms showing features that deviate from a typical peak shape but are still included in the integration because they clearly lie above the expected baseline level (blue line).

Example 2.6 (Back to Basic Rules)

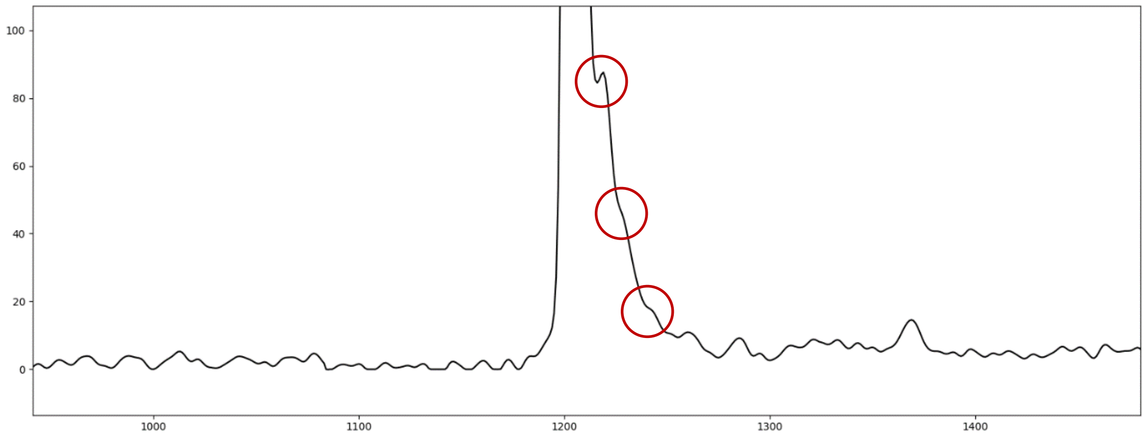

Figure 14: Electropherogram showing a peak with three potential shoulders. The first begins at a clear local minimum and is therefore classified as a shoulder. The other two do not show a distinct local minimum or zero-slope inflection and cannot be confirmed as shoulders without additional information. To verify these features, comparison with electropherograms from the same series can provide insights into whether and where a perpendicular should be dropped (see Figure 15).

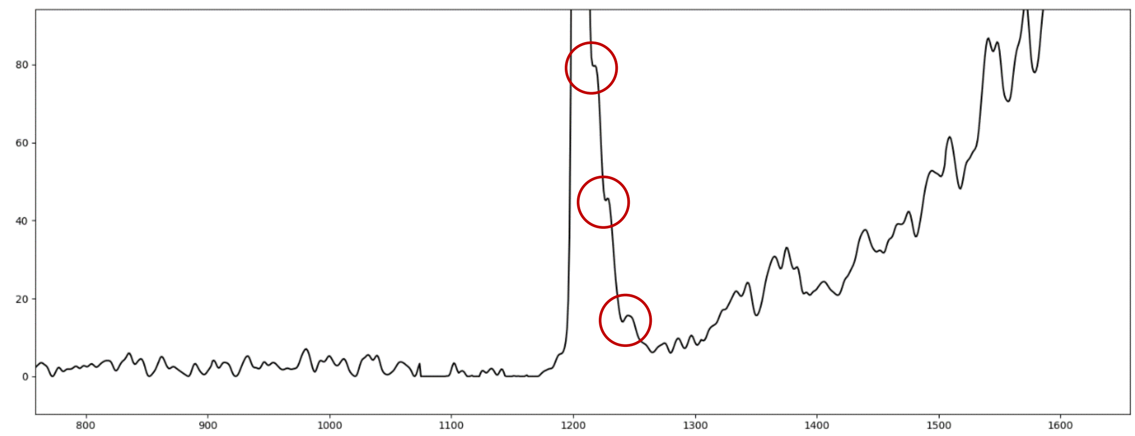

Figure 15: Electropherogram of the same series as the one in Figure 14, showing three distinct shoulders.

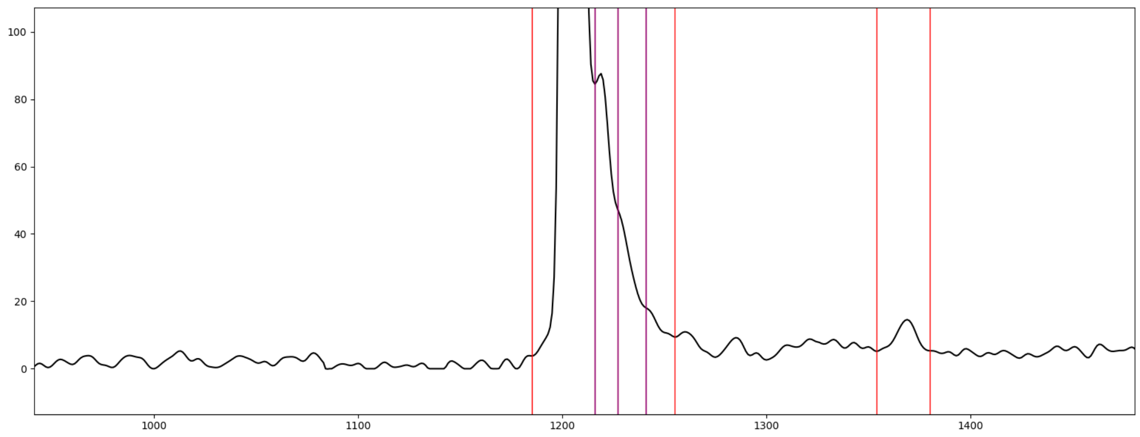

Figure 16: Electropherogram from Figure 14 with three integrated shoulders. The questionable shoulders in Figure 14 have been confirmed as shoulders based on the comparison with the electropherogram in Figure 15.

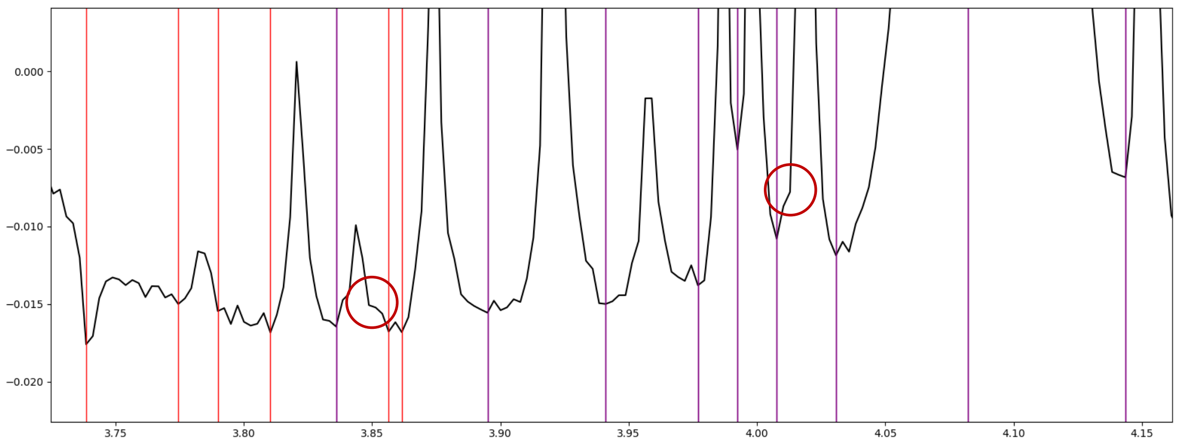

Figure 17: Electropherogram with areas that were not classified as shoulders after comparison with the background noise. The shoulder-like notches may have been caused by noise.

Example 2.7 (Back to Basic Rules)

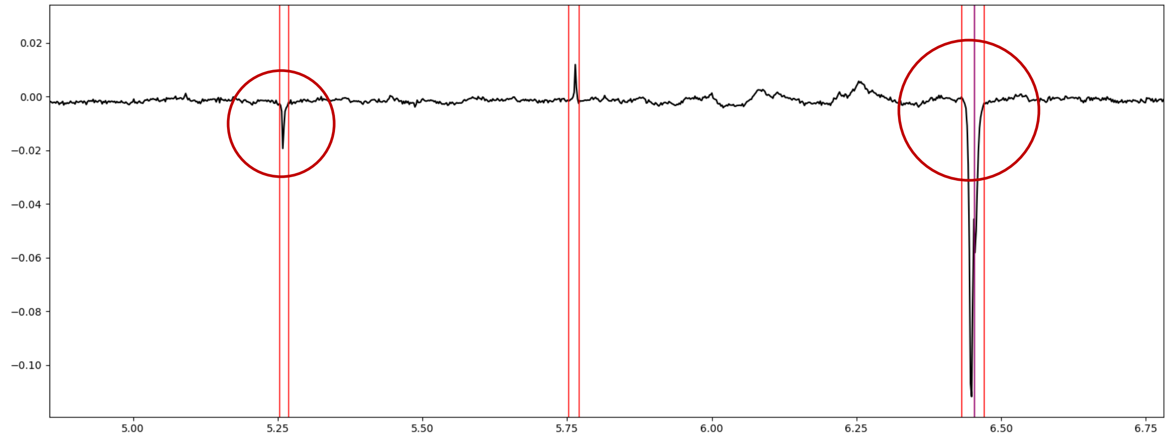

Figure 18: Electropherogram with distinct negative peaks.

Example 2.8 (Back to Basic Rules)

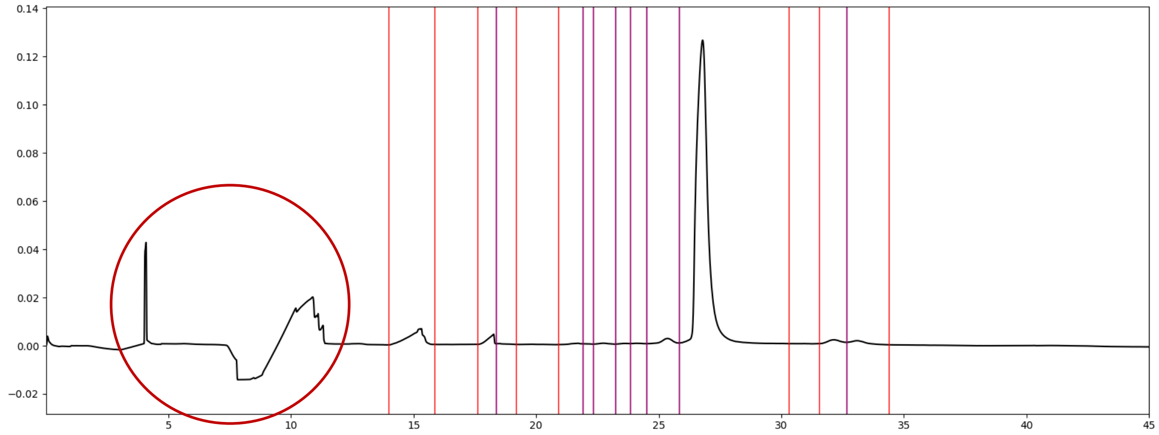

Figure 19: Electropherogram with peaks that are typical for the system used but are clearly not related to the sample of interest. These system peaks are not taken into account during integration. Blank injections can be used to confirm the occurrence of system peaks.

3 Specific Rules - Examples

Example 3.1 (Back to Rule 3.1)

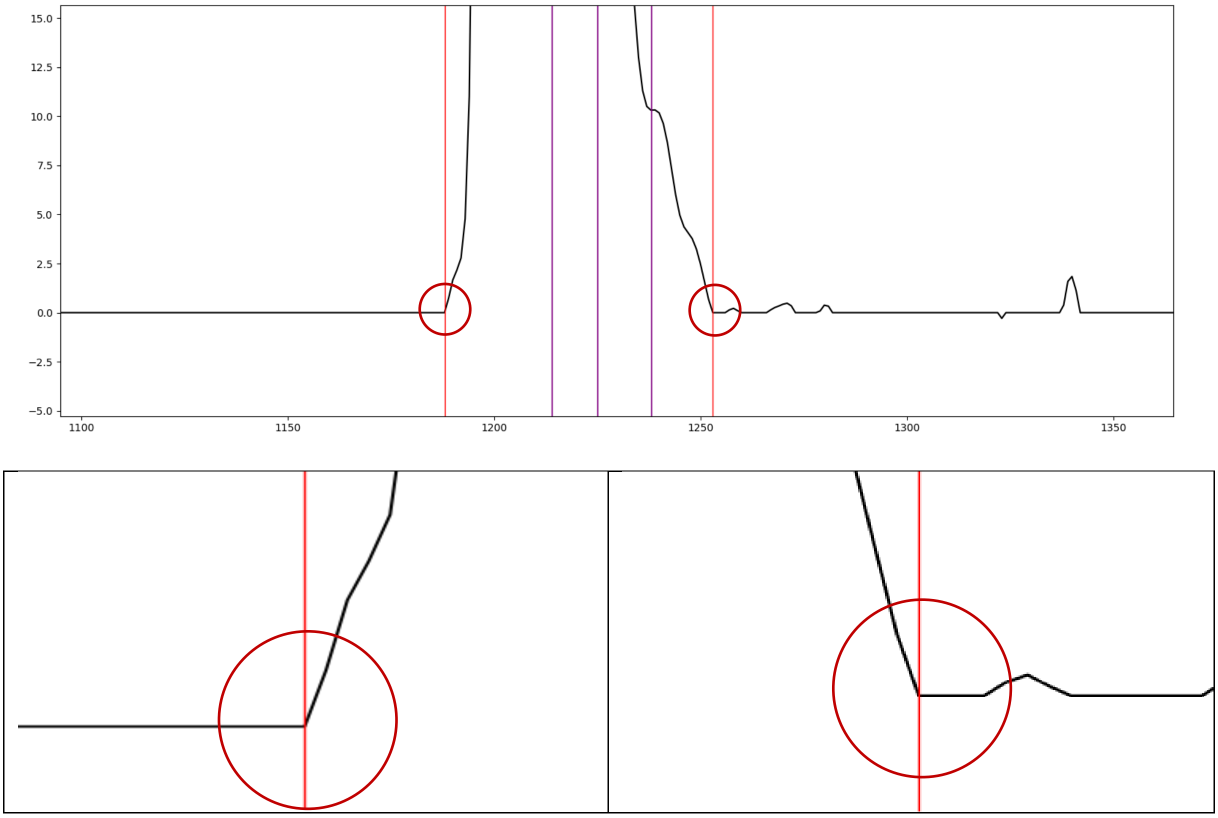

Figure 20: Integration limits set at the start and end of peaks that emerge from a flat baseline.

Example 3.2 (Back to Rule 3.2)

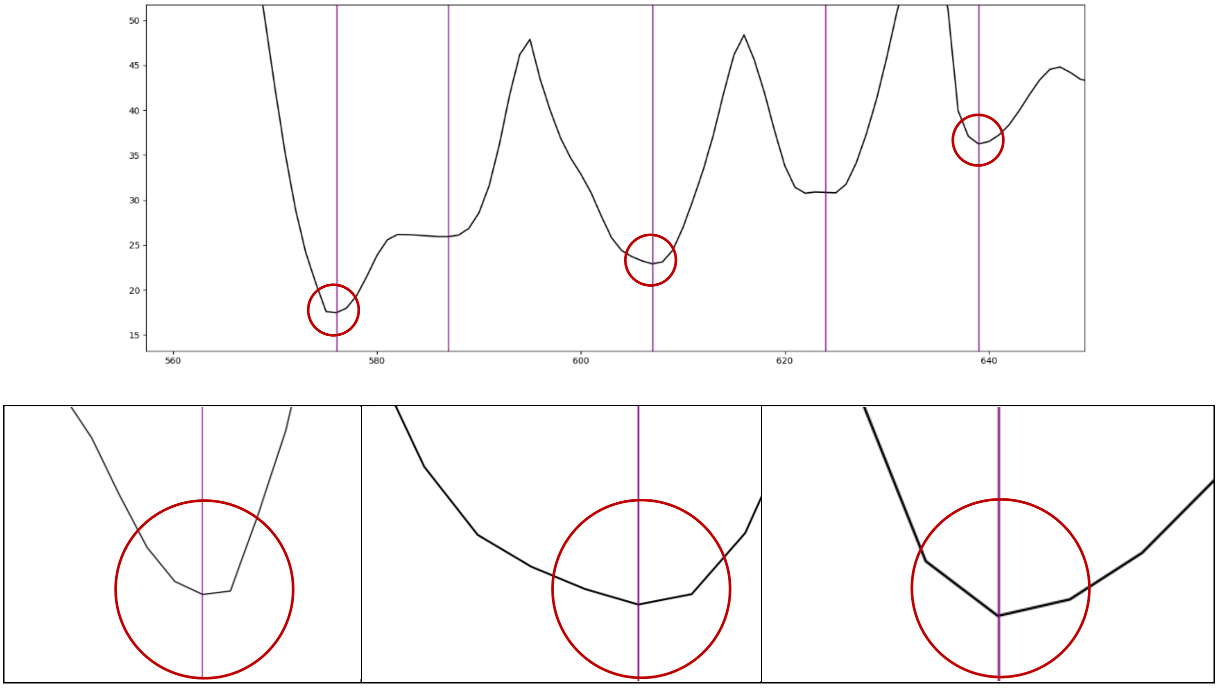

Figure 21: Shared integration limits set in the valleys between the merging peaks.

Example 3.3 (Back to Rule 3.2)

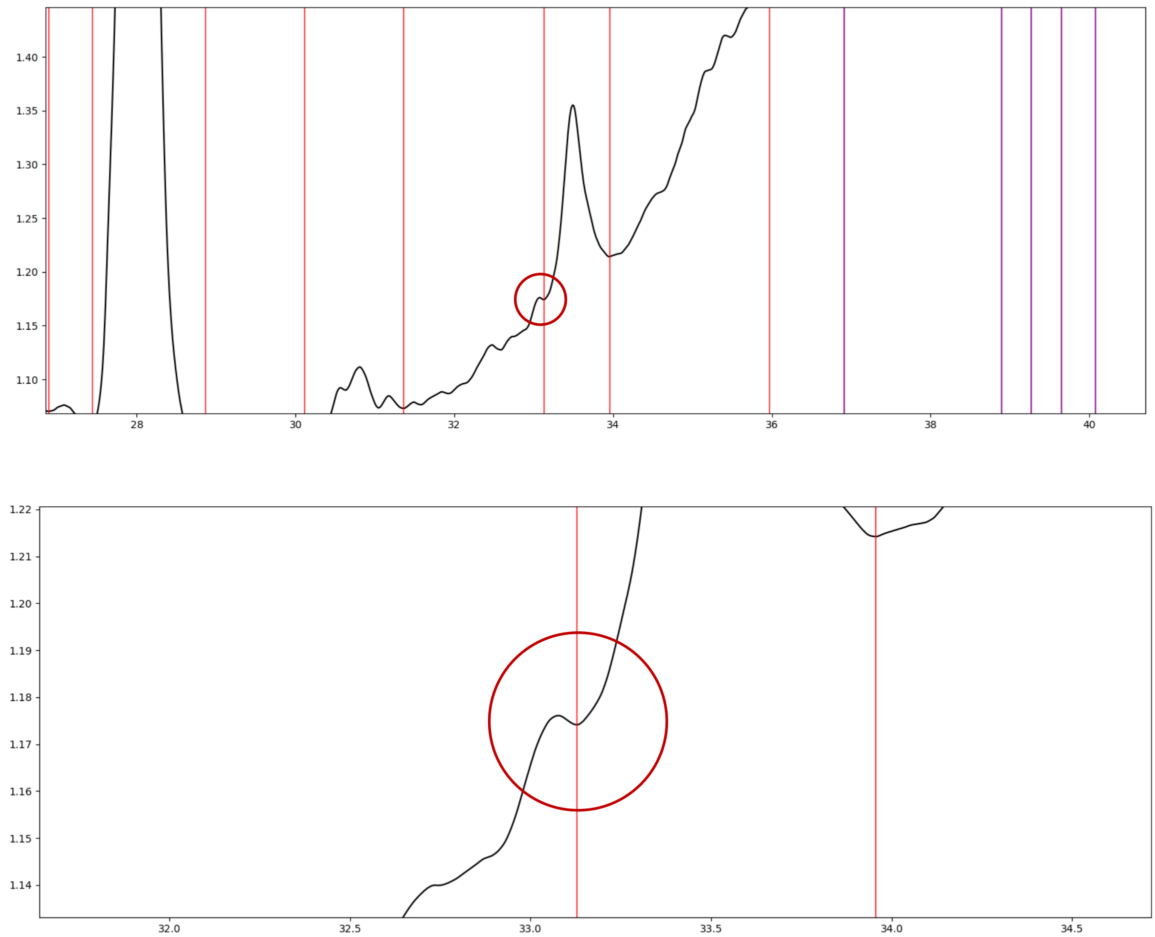

Figure 22: Integration limit set at the local minimum immediately before the peak.

Example 3.4 (Back to Rule 3.2)

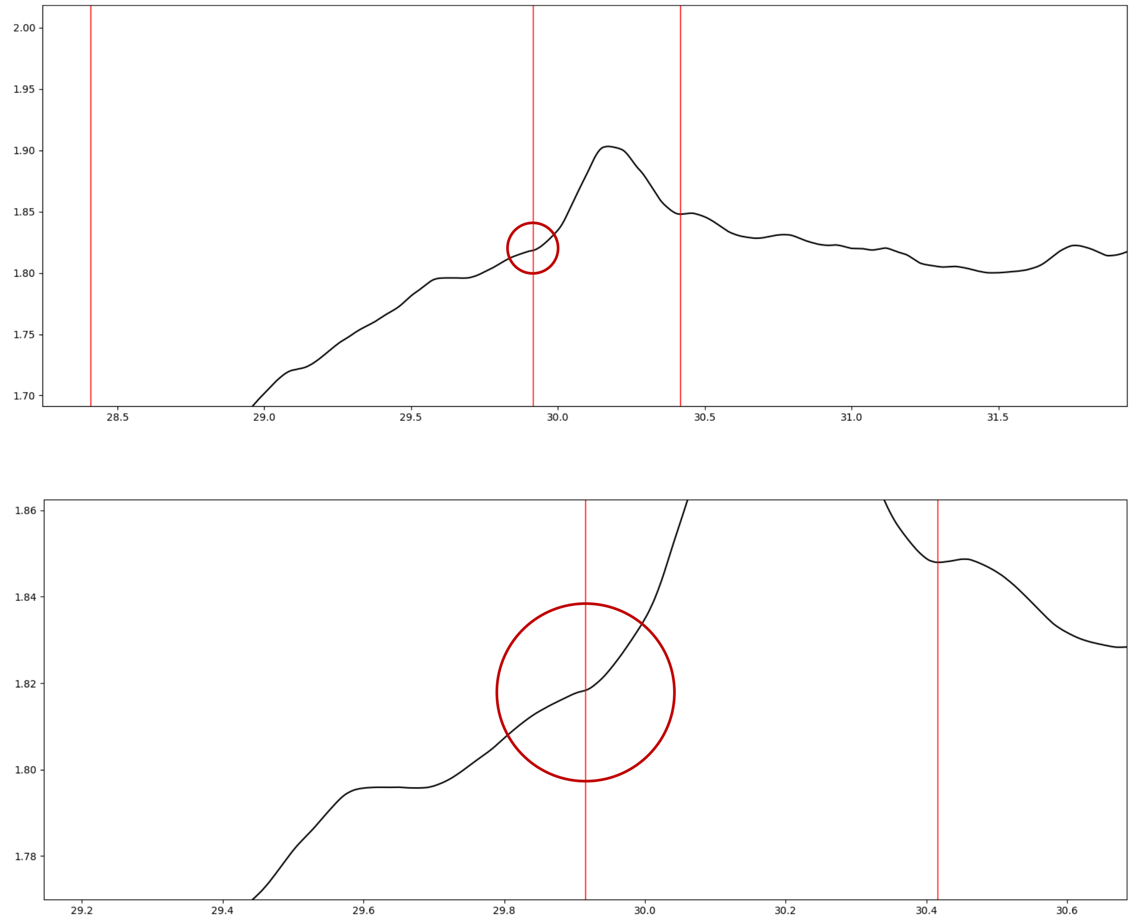

Figure 23: Integration limit set at the inflection point at the beginning of the main ascent.

Example 3.5 (Back to Rule 3.2)

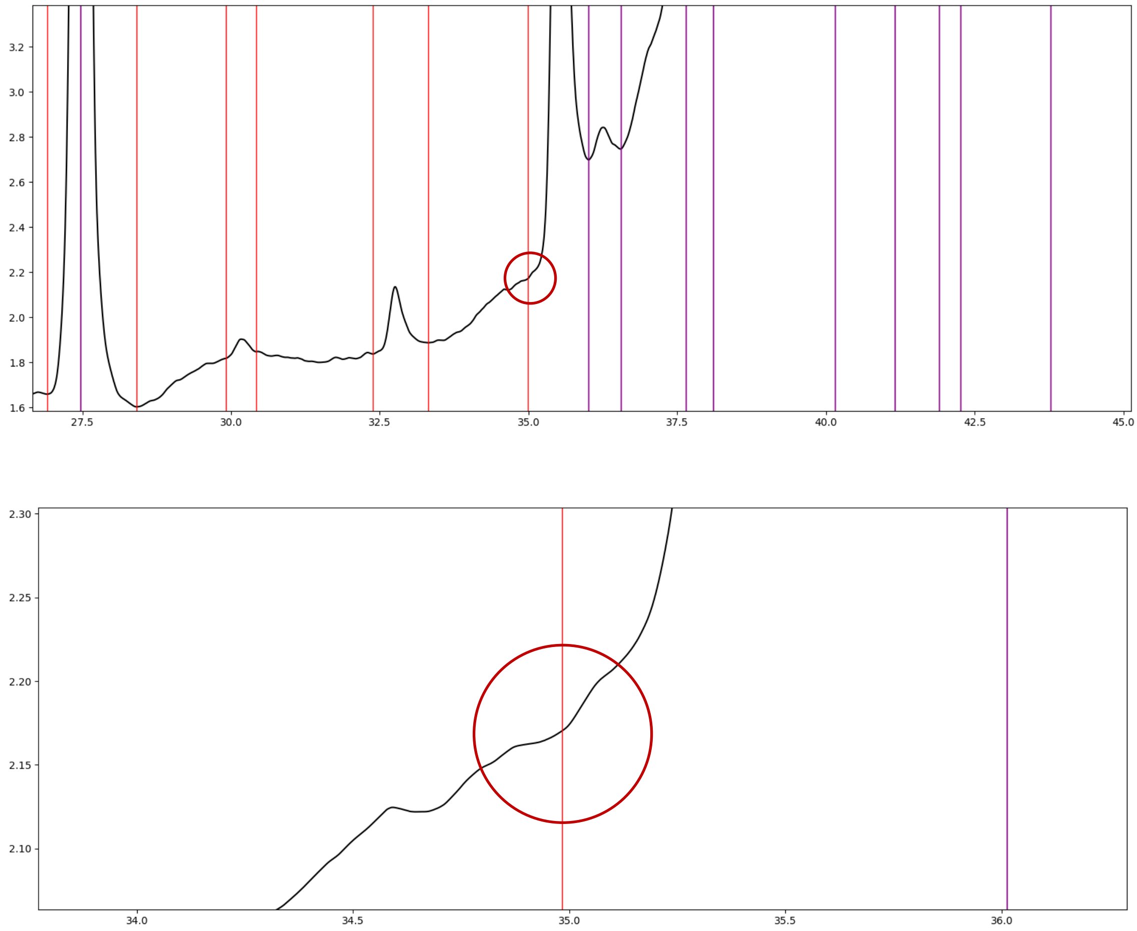

Figure 24: Integration limit set at the point at which the positive slope in the peak flank changes significantly.

Example 3.6 (Back to Rule 3.3)

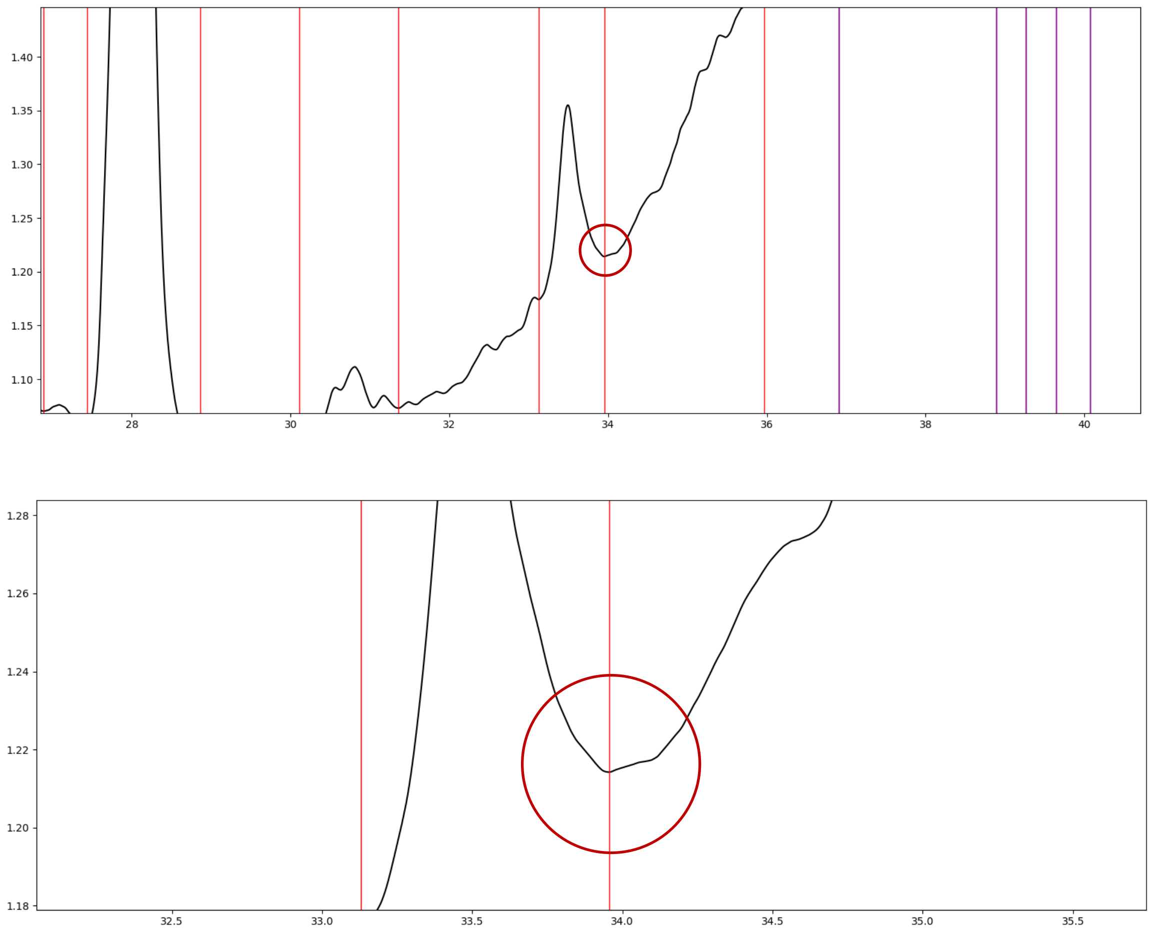

Figure 25: Integration limit set at the local minimum immediately after the peak.

Example 3.7 ([Back to Rule 3.3](#))

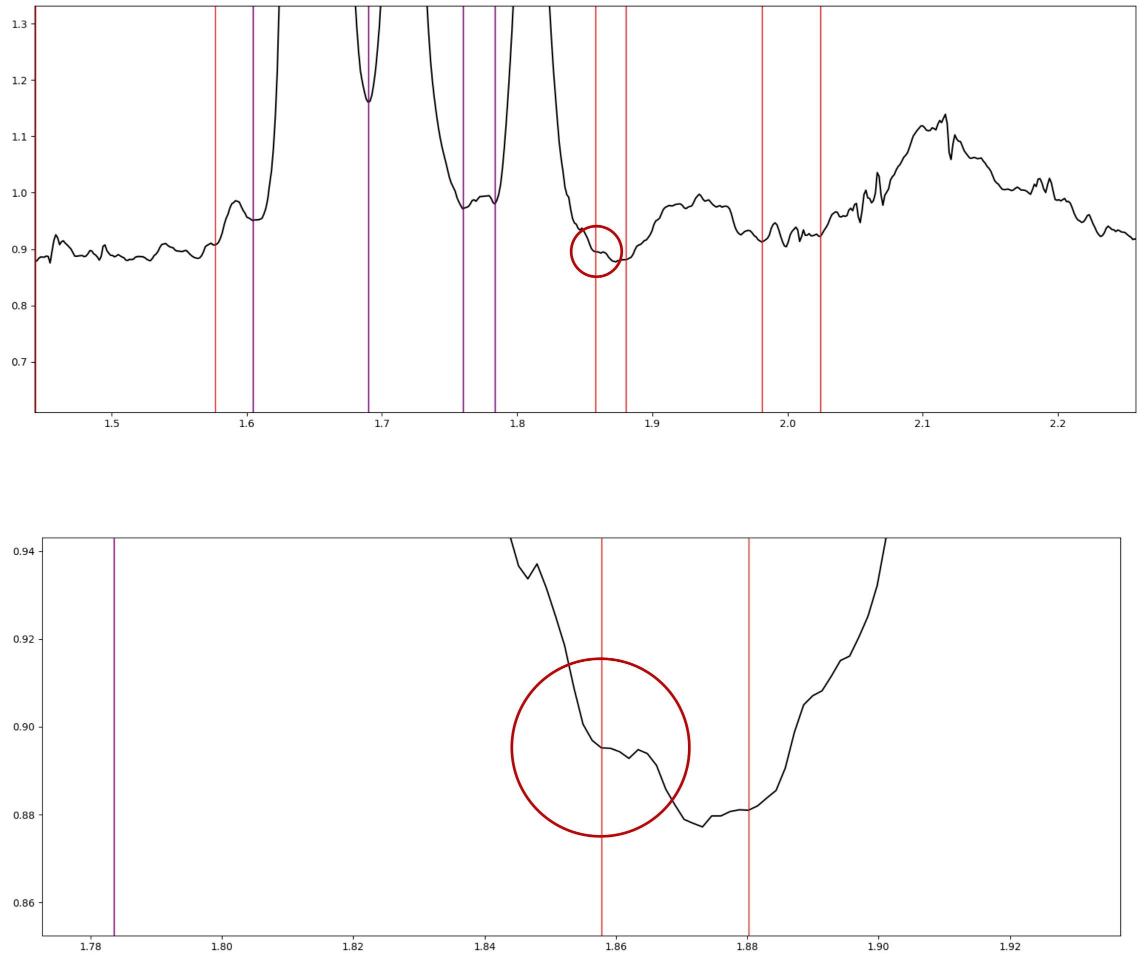

Figure 26: Integration limit set at the inflection point at the end of the main decline.

Example 3.8 (Back to Rule 3.3)

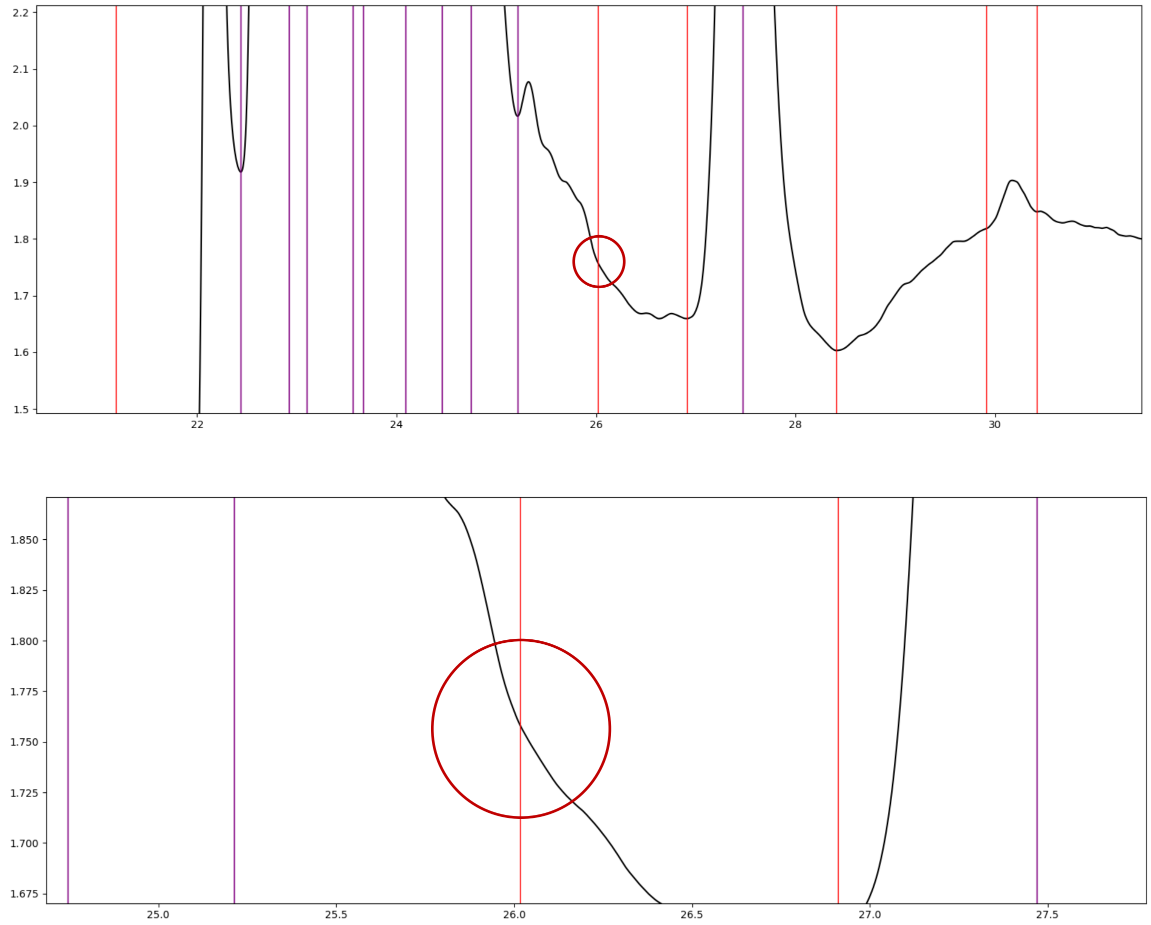

Figure 27: Integration limit set at the point at which the negative slope in the peak flank changes significantly.

Example 3.9 ([Back to Rule 3.4])

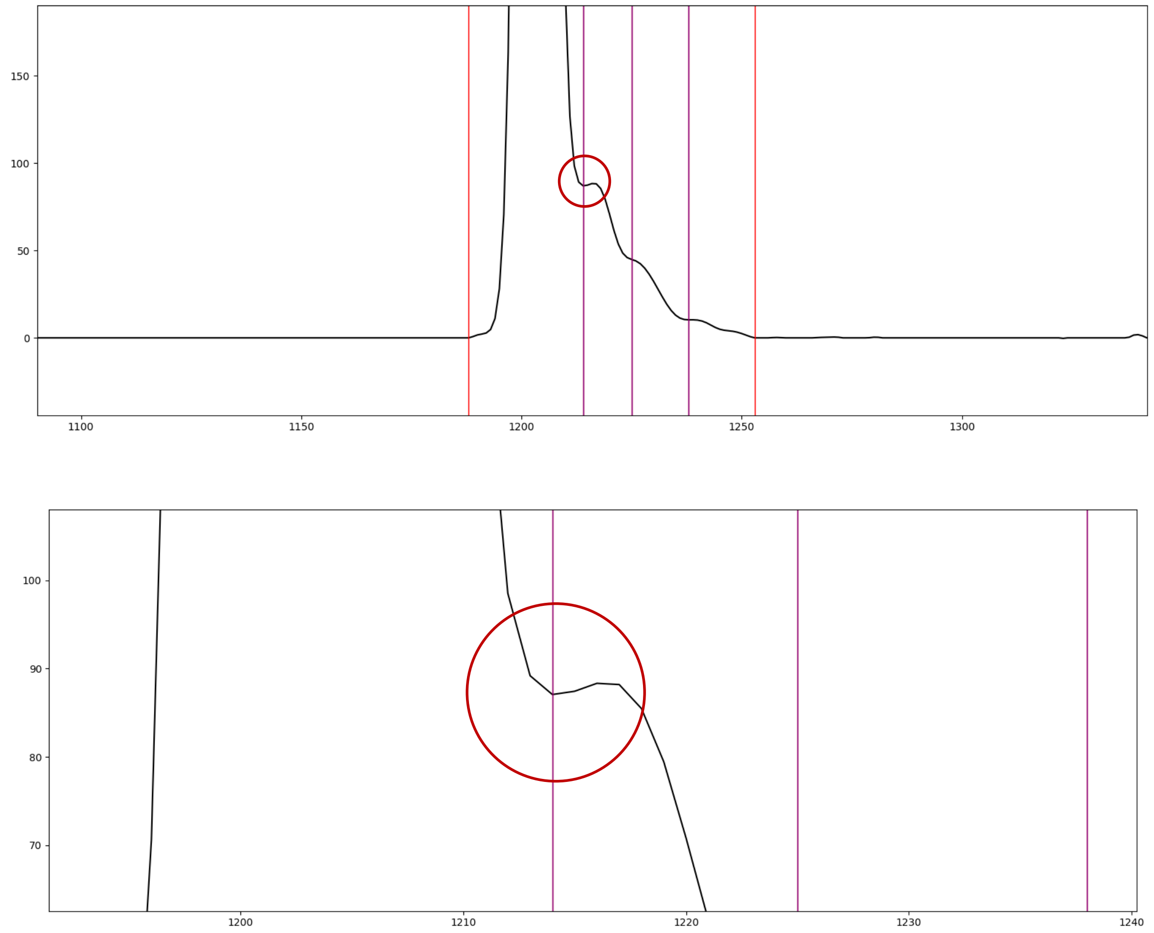

Figure 28: Integration limit set at the local minimum between main peak and shoulder.

Example 3.10 (Back to Rule 3.4)

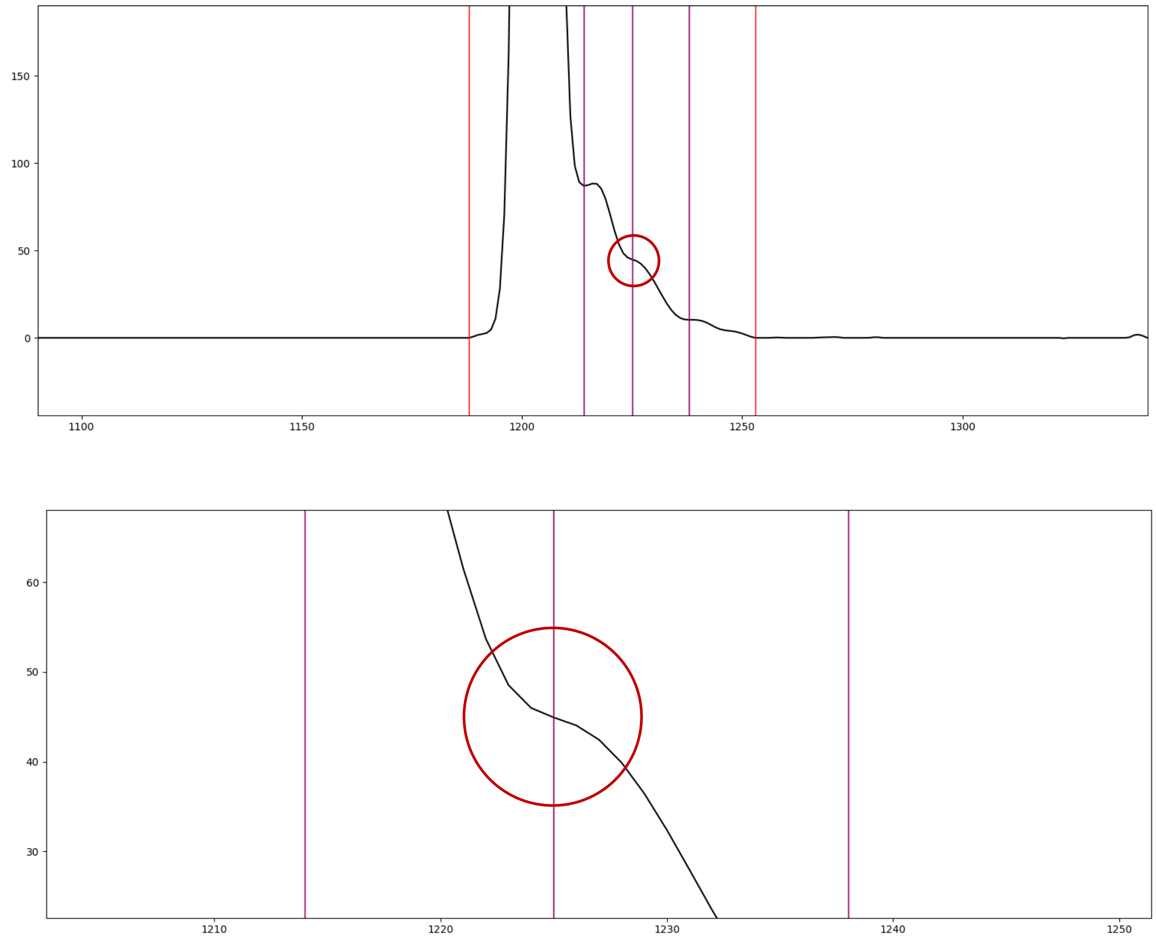

Figure 29: Integration limit set at the inflection point, which marks the beginning of the following shoulder.

Example 3.11 (Back to Rule 3.4)

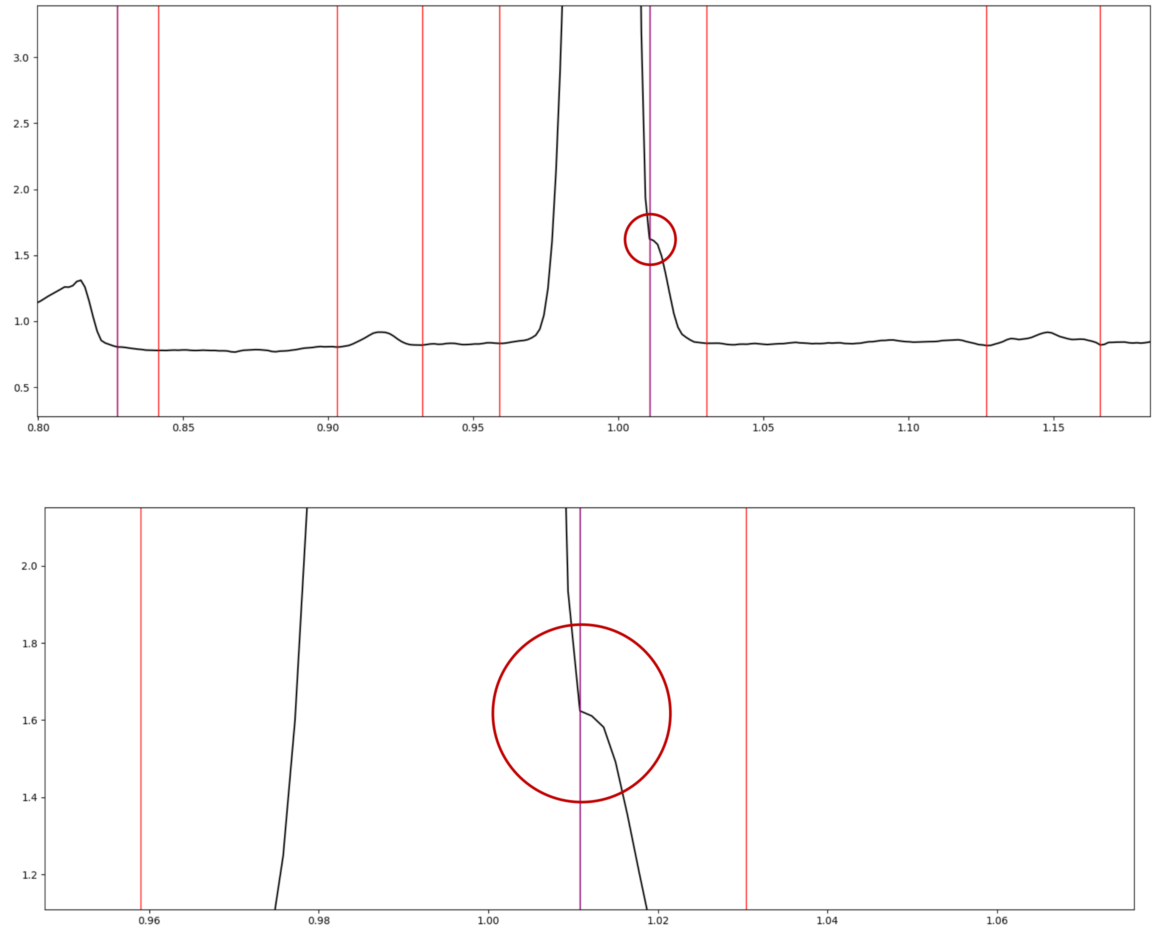

Figure 30: Integration limit set at the point at which the main descent significantly changes its rate to transition to the following shoulder. This rule can also be applied when starting with a distinct plateau.

Example 3.12 (Back to Rule 3.5)

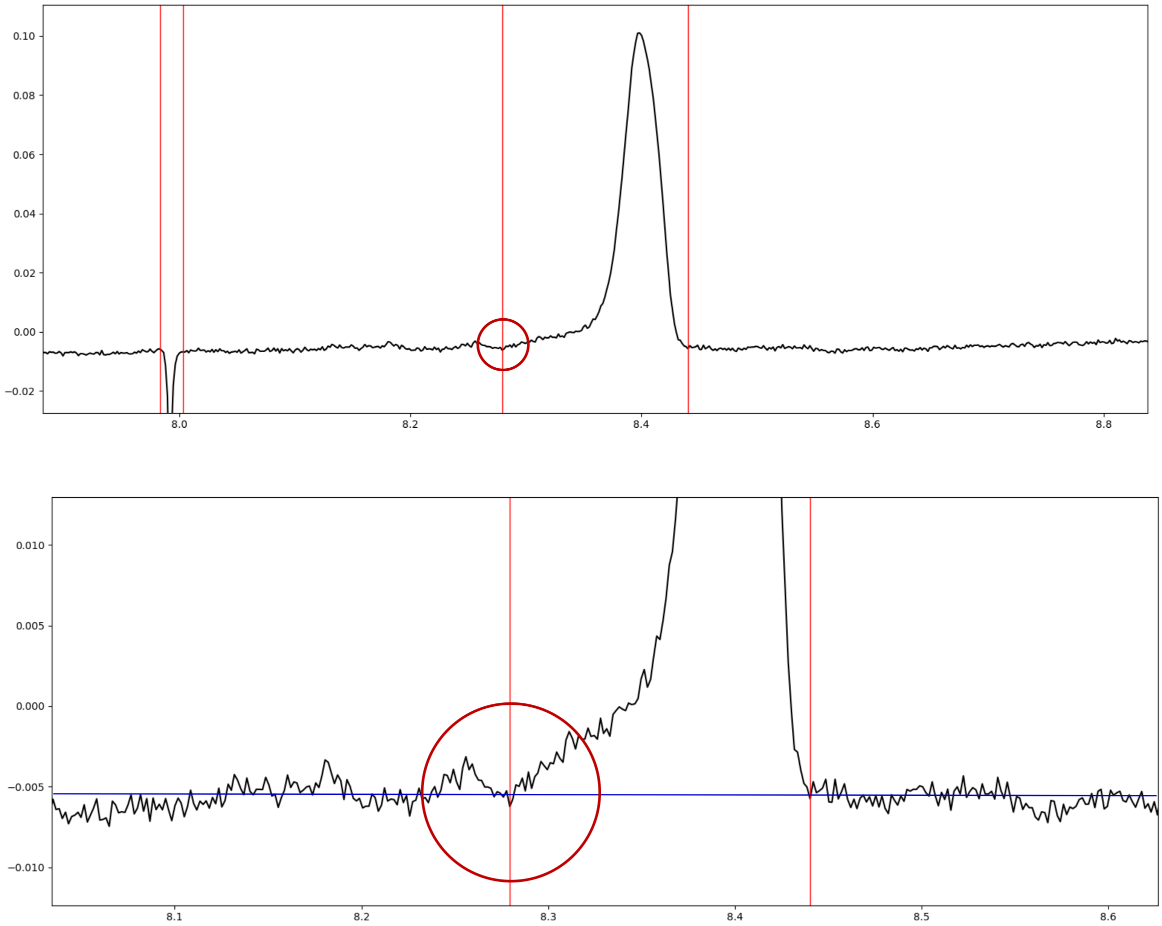

Figure 31: Integration limit set at the start of the first peak that ends above the expected course of the baseline (blue).

Example 3.13 (Back to Rule 3.6)

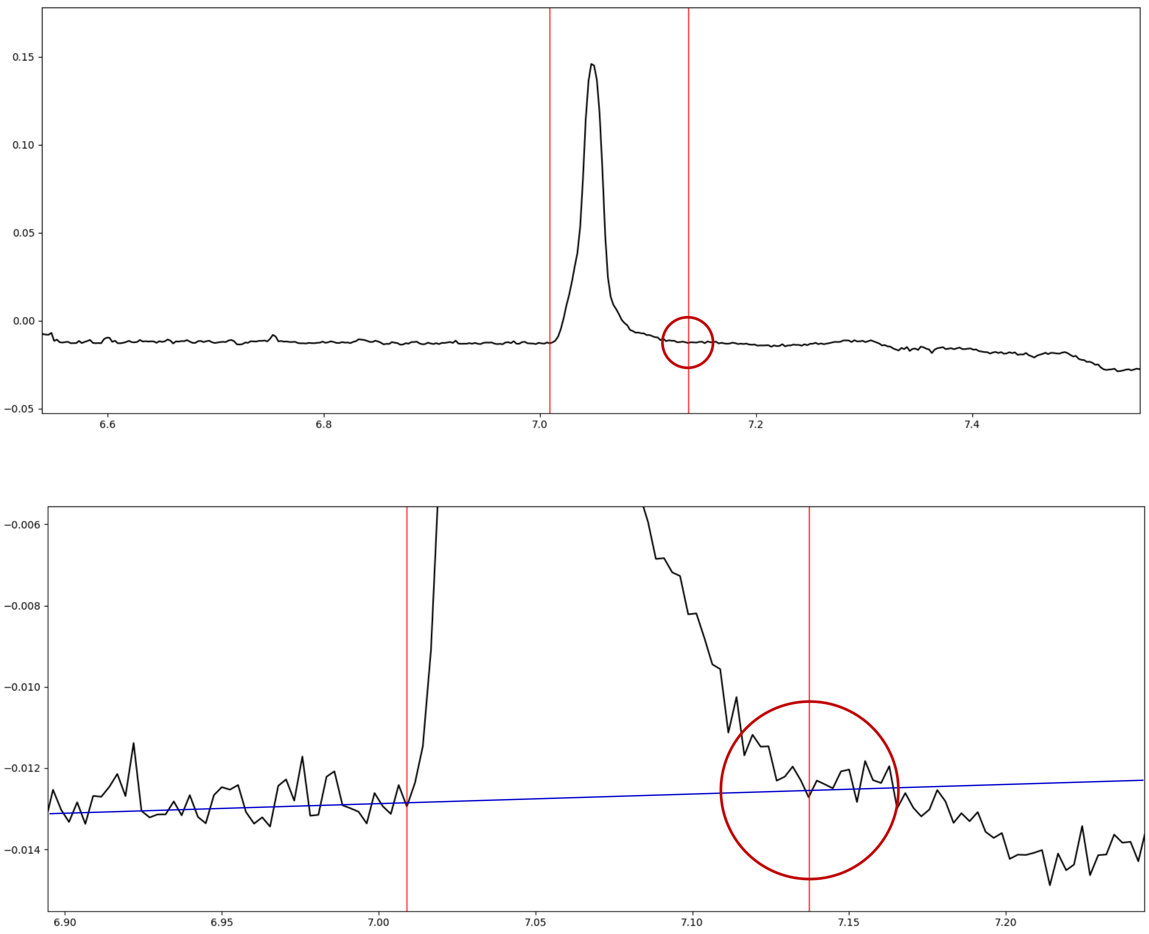

Figure 32: Integration limit set at the end of the first peak that ends below the expected course of the baseline (blue).

Example 3.14 (Back to Rule 3.7)

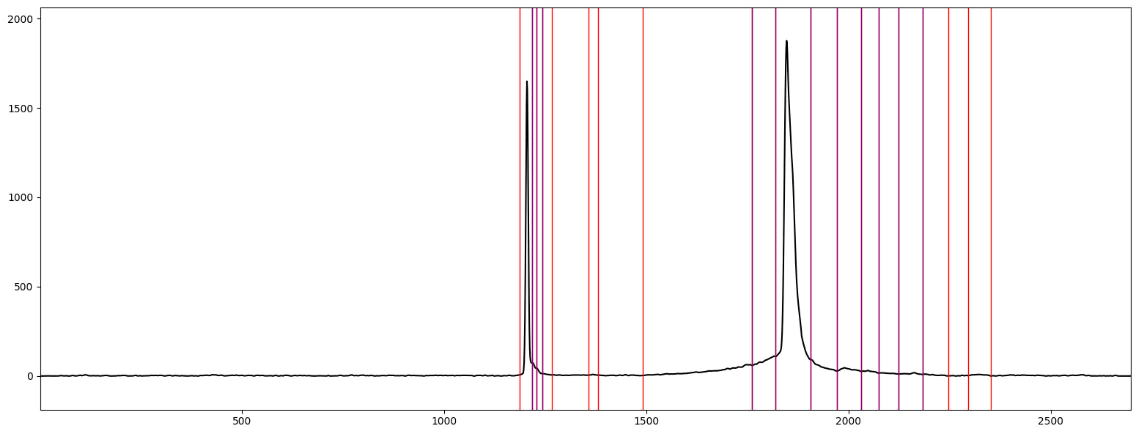

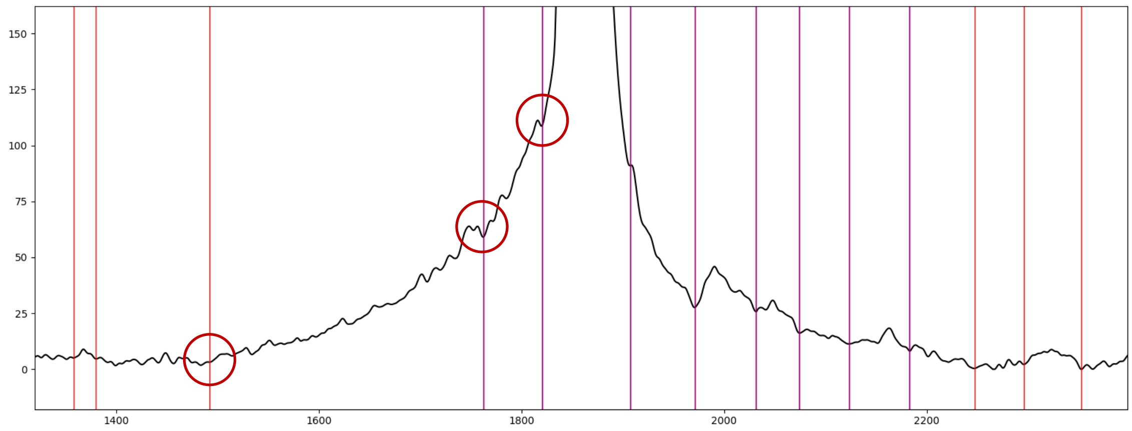

Figure 33: Integration limits set at the start of the front-like series of unresolved peaks/shoulders, at the transition between subunits of the front-like area as well as at the transition to the main peak.

Example 3.15 (Back to Rule 3.7)

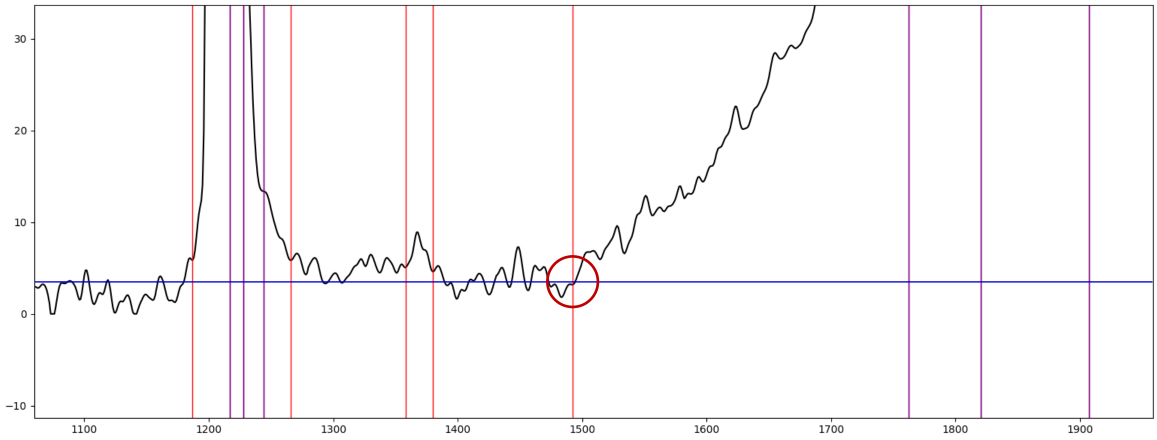

Figure 34: Integration limit set at the start of the first peak that ends above the expected course of the baseline (blue). This peak will be considered the beginning of the front-like series of unresolved peaks/shoulders.

Example 3.16 (Back to Rule 3.7)

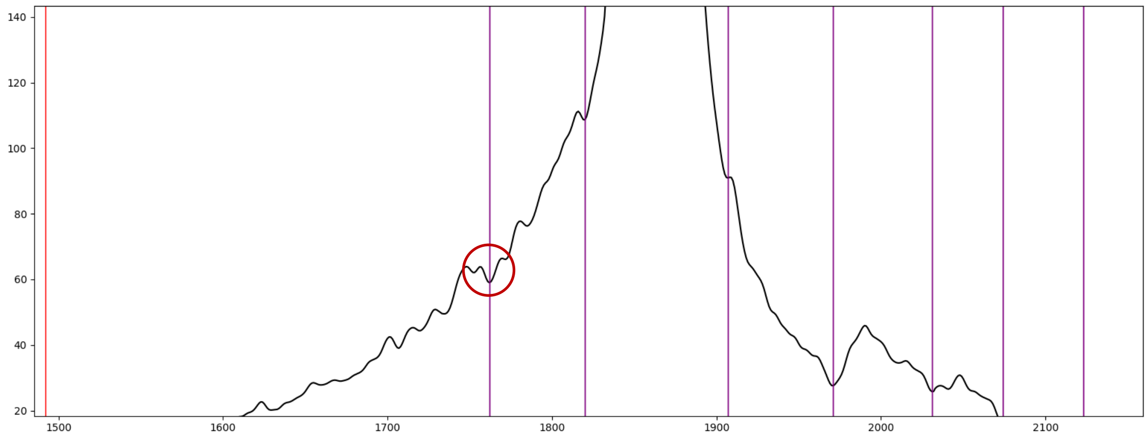

Figure 35: Integration limit set at the transition between two subunits of the front-like area.

Example 3.17 (Back to Rule 3.7)

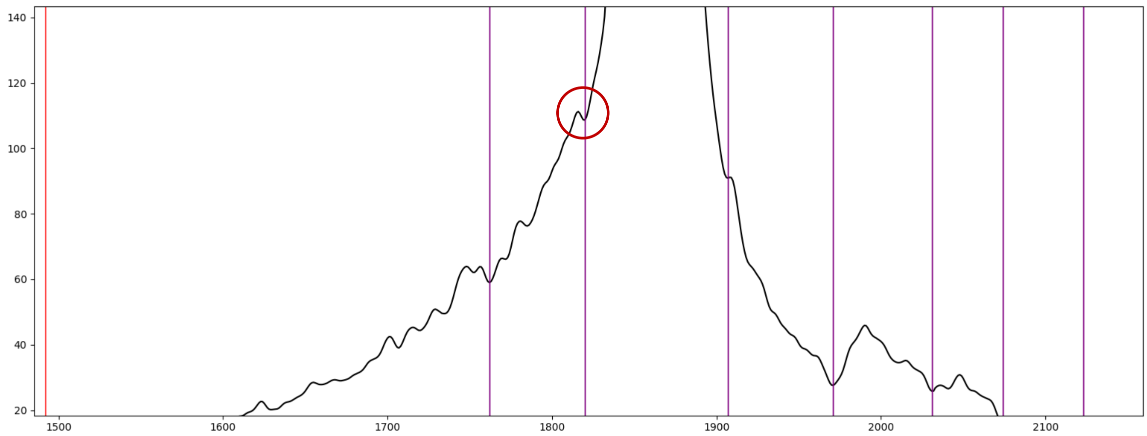

Figure 36: Integration limit set at the transition from the front-like area to the main peak.

Example 3.18 (Back to Rule 3.8)

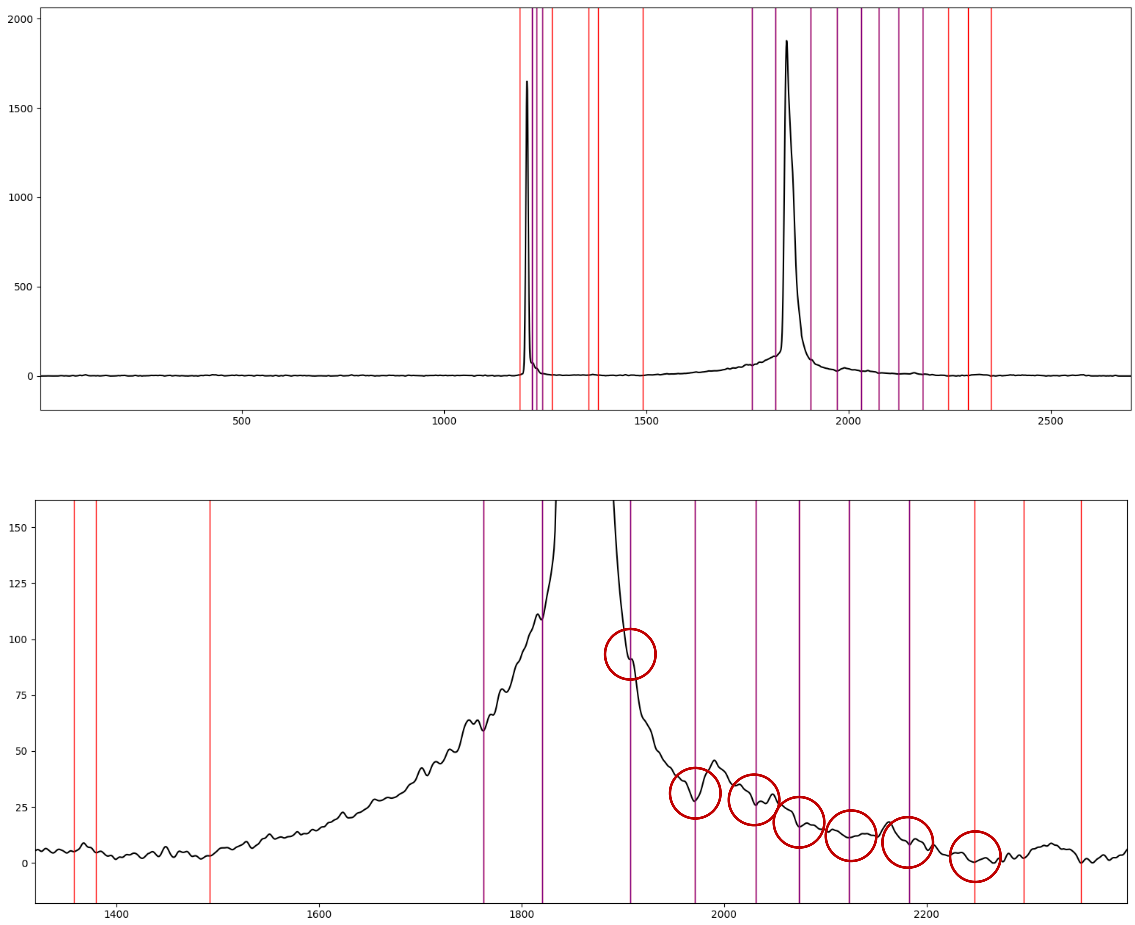

Figure 37: Integration limits set at the transition from the main peak, at the transition between subunits of the tail-like series of unresolved peaks/shoulders as well as at the end of the tail-like area.

Example 3.19 (Back to Rule 3.8)

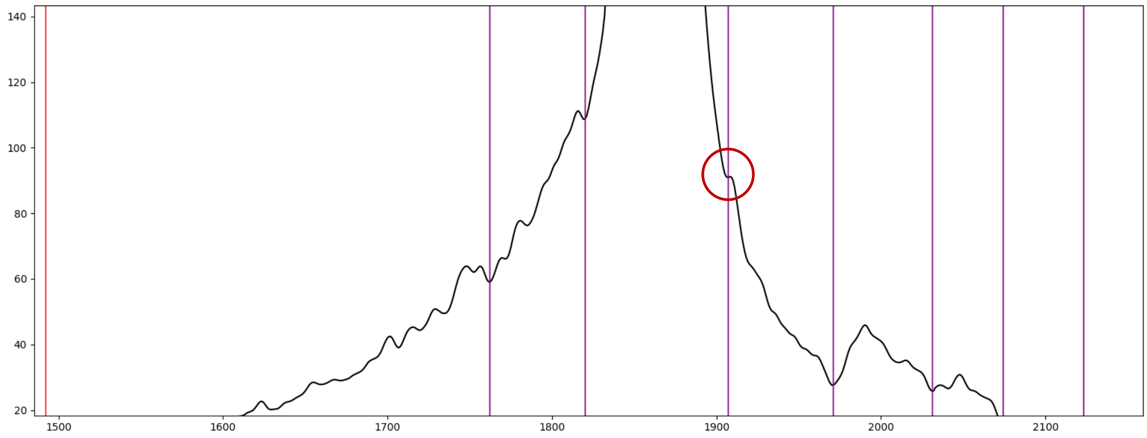

Figure 38: Integration limit set at the transition from the main peak to the tail-like area.

Example 3.20 (Back to Rule 3.8)

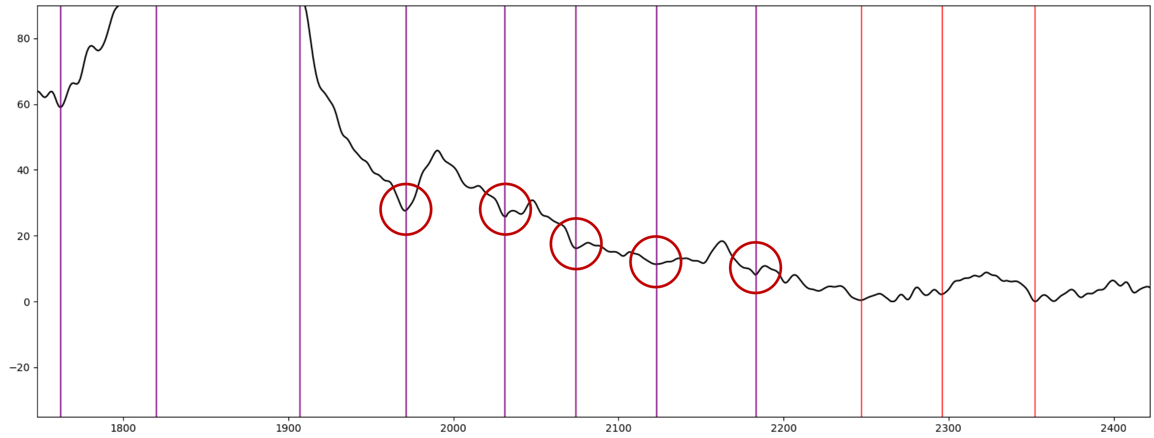

Figure 39: Integration limits set at the transitions between several subunits of the tail-like area.

Example 3.21 (Back to Rule 3.8)

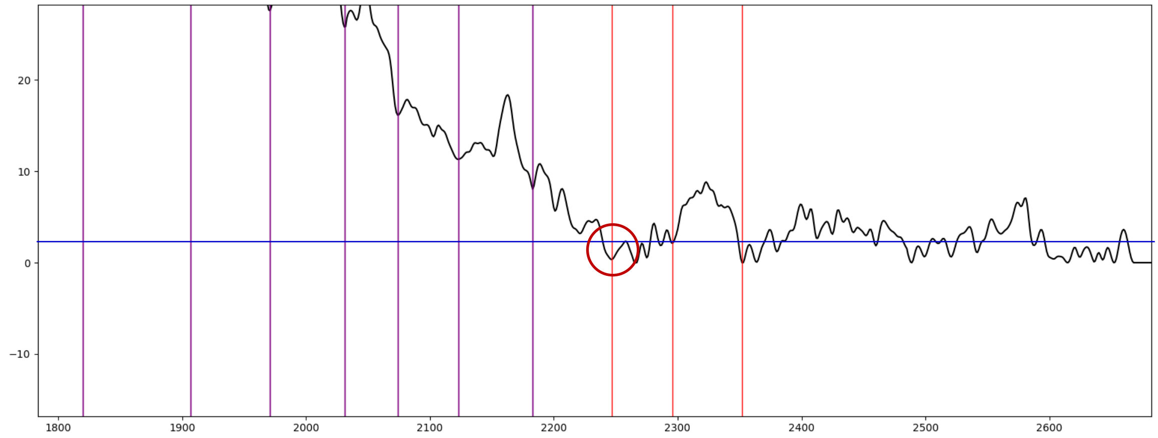

Figure 40: Integration limit set at the end of the first peak that ends below the expected course of the baseline (blue). This peak will be considered the end of the tail-like series of unresolved peaks/shoulders.

Example 3.22 (Back to Rule 3.9)

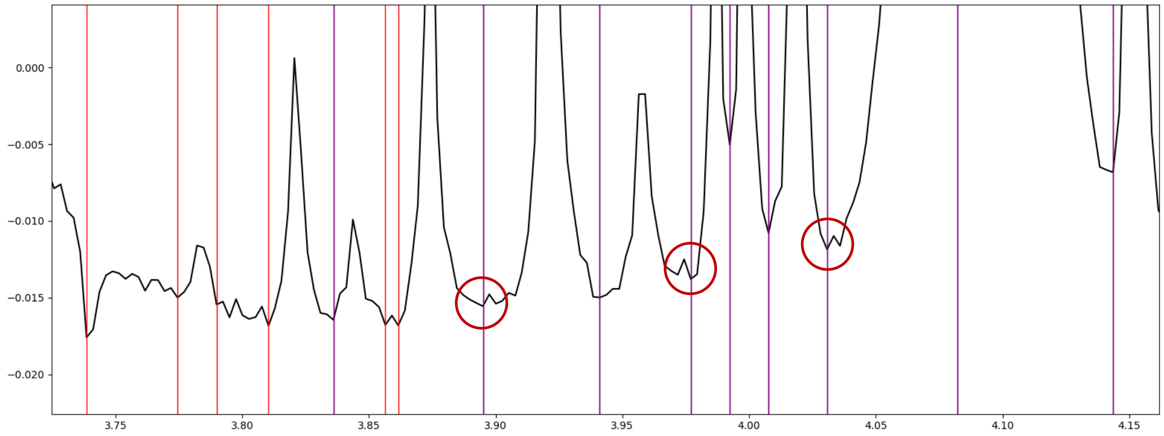

Figure 41: Integration limits set at the lower end of noise peaks which overlay the valleys between unresolved peaks.

Example 3.23 (Back to Rule 3.10)

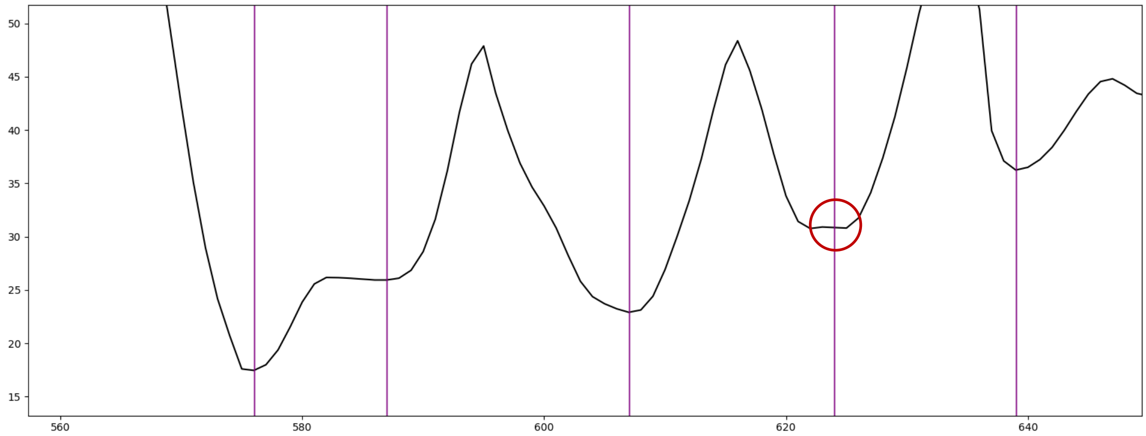

Figure 42: Integration limit set at a bridging valley between two unresolved peaks.

Example 3.24 (Back to Rule 3.11)

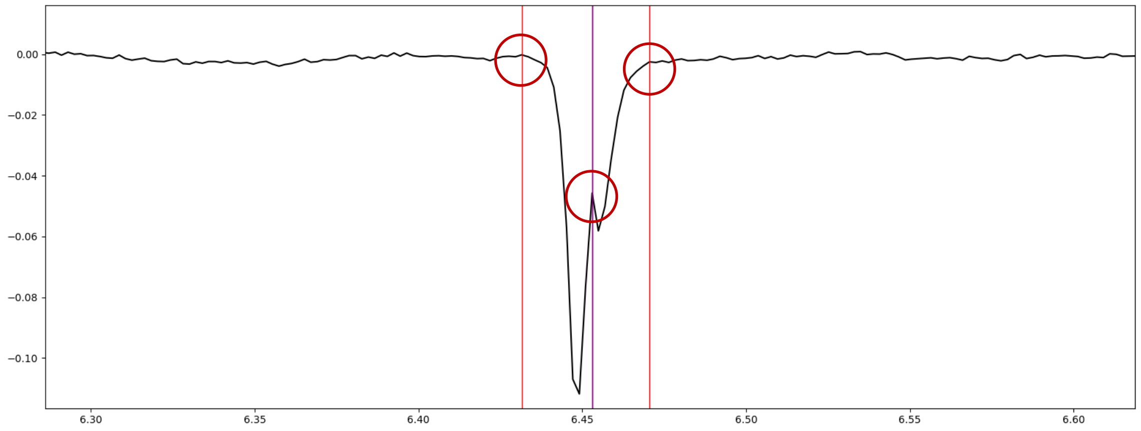

Figure 43: Integration limits set in accordance to the reversed rules from positive peaks due to integration of a negative peak.

Example 3.25 (Back to Rule 3.12)

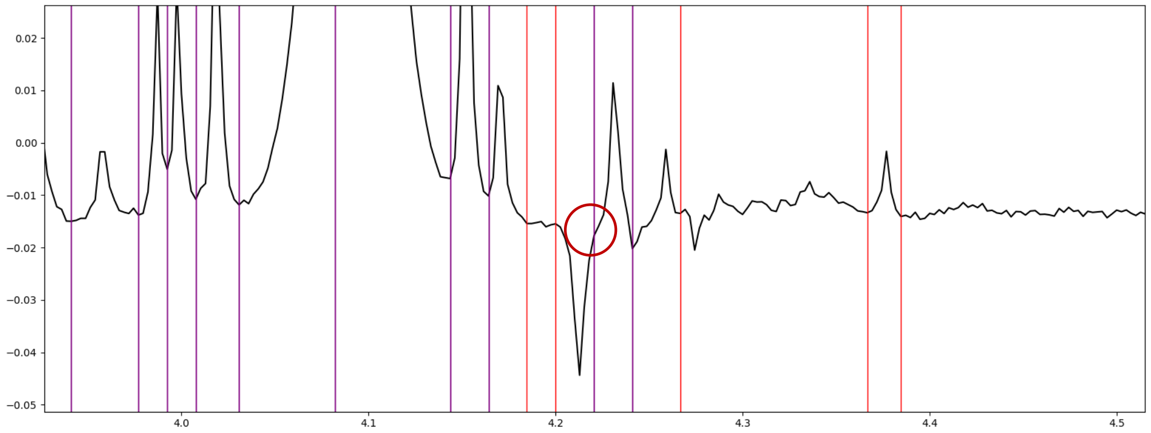

Figure 44: Integration limit set at the inflection between a negative and a merging positive peak.
